# Upregulation of interferon-γ activation in patients with anti-interferon-γ autoantibodies immunodeficiency syndrome: insights from single-cell analysis

**DOI:** 10.3389/fimmu.2025.1659383

**Published:** 2026-02-03

**Authors:** Si-qiao Liang, Xue-mei Huang, Xiao-na Liang, Si-yao Wu, Li-mei Hong, Ni Chen, Zeng-tao Luo, Yan Ning, Meng-chan Wang, Zhi-yi He

**Affiliations:** Department of Respiratory and Critical Care Medicine, The First Affiliated Hospital of Guangxi Medical University, Nanning, Guangxi, China

**Keywords:** anti-interferon-γ autoantibodies, B cell, immune characteristics, single-cell RNA sequencing, T cell

## Abstract

**Background:**

Anti-interferon-γ autoantibodies (AIGAs) immunodeficiency syndrome is an emerging adult-onset immunodeficiency causing opportunistic infections. However, its comprehensive immune landscape remains elusive. This study presents the first single-cell RNA sequencing (scRNA-seq) analysis of AIGAs immunodeficiency syndrome, aiming to delineate its pathogenic mechanisms.

**Methods:**

We performed scRNA-seq on peripheral blood mononuclear cells (PBMCs) from 8 AIGAs immunodeficiency syndrome patients (4 infective, 4 stable phase) and 3 healthy controls. Findings were validated by flow cytometry in an expanded cohort (15 patients vs. 10 controls).

**Results:**

Single-cell RNA sequencing of PBMCs from patients with AIGAs immunodeficiency syndrome identified a comprehensive immune subset profile, including effector memory CD4^+^ T cells, naive CD4^+^ T cells, regulatory T cells, GNLY^+^ CD8^+^ Tem, GZMK^+^ CD8^+^ Tem, naive CD8^+^ T cells, naive B cells, memory B cells, plasma cells, ISG^+^ atypical B cells, monocytes, and NKT cells. ScRNA-seq analysis revealed a significantly higher proportion of Th1 cells (16.62% vs. 6.94% in controls) and ISG^+^ B cells (2.95% vs. 0.53%), alongside a lower proportion of plasma cells (9.30% vs. 17.79%) and memory B cells (9.54% vs. 27.35%). Flow cytometry consistently confirmed the increase in Th1 cells (21.84% [14.87–27.57] vs. 11.96% [7.19–15.74]) and decreases in marginal zone B cells (2.87% [1.71–4.45] vs. 8.60% [6.77–15.65]), memory B cells (13.85% [5.72–20.23] vs. 22.96% [16.39–33.83]), and class-switched B cells (6.11% [2.39–9.10] vs. 10.18% [5.35–15.77]). Transcriptome analysis demonstrated upregulated expression of interferon-response and HLA genes (e.g., HLA-DQB1, HLA-DQA1, HLA-DRB1), whereas IRF1 was downregulated across all subsets; functional enrichment analyses further highlighted significant activation in IFN signaling and B cell activation pathways. CellChat and pseudotime analyses indicated that CD4^+^ Tem and CD14^+^ monocytes drive sustained Th1 inflammation and monocyte hyperactivation through enhanced pro-inflammatory and antigen-presenting interactions, with T-cell differentiation skewed toward terminal effectors and B-cell development disrupted by ISG^+^ B cell emergence, premature plasma cell formation, and IGLC3-biased class switching, collectively delineating the interferon-mediated immunopathology of AIGAs immunodeficiency syndrome.

**Conclusions:**

In summary, this first single-cell atlas maps AIGAs immunodeficiency syndrome as a Th1-skewed, IFN-γ-driven disorder sustained by CD4^+^ Tem–CD14^+^ monocyte crosstalk. It combines T-cell activation, expanded Th1 and ISG^+^ B cells, and loss of memory/plasma B cells to drive autoantibody generation. Skewed T- and B-cell trajectories and polygenic up-regulation of interferon/HLA genes provide a clear mechanistic rationale for targeted therapy.

## Introduction

Anti-interferon-γ autoantibodies (AIGAs) immunodeficiency syndrome is an emerging immunodeficiency disorder primarily affecting adults, characterized by opportunistic disseminated infections due to neutralizing AIGAs (1, 2). Interferon-gamma (IFN-γ), which is mainly secreted by macrophages, CD4^+^ T cells, CD8^+^ T cells, and natural killer (NK) cells, plays a crucial role in immune responses and defense against intracellular pathogen infections (3). Signaling by IFN-γ to IFN-γ receptor (IFNGR), can activate the Janus kinase (JAK)-signal transducer and activator of transcription 1 (STAT1) pathway, which subsequently leads to the expression of interferon-stimulated genes (ISGs) (3, 4). Neutralizing AIGAs block IFN-γ binding, inhibiting STAT-1 phosphorylation and downstream biological effects, such as downregulation of the expression of TNF-α and IL-12 (1, 5). AIGAs impede IFN-γ-mediated antimicrobial immunity, particularly impairing macrophage activation against intracellular pathogens (6).

Recent studies (2, 7, 8), including our latest research (9), have confirmed that AIGAs immunodeficiency syndrome is frequently accompanied by recurrent intracellular infections. Despite receiving appropriate antibiotic treatment, patients still experience a relatively high level of fatality (2, 10). These outcomes are closely linked to immunodeficiency caused by AIGAs. Cumulative research reports that this immunodeficiency disorder has been linked to diverse factors, such as genetic variations, immune responses, and pathogen infections.

The distribution of AIGAs immunodeficiency syndrome is regional, mainly in Southeast Asia, including China, Vietnam, and other areas (7). In China, it is predominantly found in Guangxi. Linkage disequilibrium between *HLA-DRB1* and *HLA-DQB1* alleles was observed in most of the AIGAs-positive patients from Southeast Asia (11–13). AIGAs, produced by B cells, are primarily IgG, with IgG1 and IgG4 being the most common subtypes (1). Study has showed that patients had higher levels of transitional and plasmablast B cells, as well as higher levels of effector memory and senescent CD8^+^ CD57^+^ T cells, and Th17 cells; while having lower levels of naive T cells and Treg cells (14). Another study indicated that patients exhibit decreased CD4^+^ T cells and increased NK cells (15). Despite advancements in research, such as the candidate gene selection, and elucidation of cell subsets, the immune characteristics and pathogenesis of AIGAs immunodeficiency syndrome remains poorly understood.

Previous immunophenotyping studies of AIGAs immunodeficiency syndrome have relied on low-throughput technologies like flow cytometry. These approaches depend on predefined surface markers, inevitably overlooking rare pathogenic subsets, subtle transitional states, and coordinated gene expression programs across cell types. In contrast, single-cell RNA sequencing (scRNA-seq) provides an unbiased, whole-transcriptome view of peripheral blood immune cells. Free from prior marker selection, scRNA-seq can not only identify novel or rare cell populations but also precisely resolve phenotypically similar states, and elucidate signaling pathways, transcriptional networks, and differentiation trajectories. This technology thus offers a higher-dimensional, functional perspective on immune dysregulation and disease heterogeneity (16). Despite its power, scRNA-seq has not yet been applied to AIGAs immunodeficiency syndrome.

In this study, we performed scRNA-seq of peripheral-blood mononuclear cells from eight patients with AIGAs immunodeficiency syndrome and three healthy controls using the 10x Genomics platform to comprehensively map the immune-cell landscape and delineate disease-specific heterogeneity. Our findings provide the first in-depth atlas of AIGAs immunodeficiency syndrome, offering novel insights into its immune characteristics and pathogenesis.

## Materials and methods

### Acquisition of the study sample

This study was approved by the Ethics Committee of The First Affiliated Hospital of Guangxi Medical University (IRB Protocol Number: 2022-KT-Guike-127), and all donors signed written informed consent. Eight AIGAs-positive patients (P1–P8) and three healthy controls (H1–H3) were enrolled for this study; all patients were from the Department of Respiratory and Critical Care Medicine, The First Affiliated Hospital of Guangxi Medical University, and were diagnosed with AIGAs immunodeficiency syndrome. Among these patients, P2, P3, P4, and P5 were in the stable phase with immune damage, while P1, P6, P7, and P8 were in the infective phase.

In the study, serum AIGAs were assessed using an indirect enzyme-linked immunosorbent assay (ELISA), while the neutralizing capacity of AIGAs was determined via Western blotting. All patients tested positive for AIGAs through ELISA, which were subsequently confirmed to possess neutralizing abilities via Western blotting.

6 mL of peripheral venous blood was collected from each sample, and PBMCs were isolated using density gradient centrifugation with Ficoll-Hypaque.

### Case definition

Infective phase was defined as patients exhibiting symptoms of infection, such as chills, fever, cough, sputum, skin, and soft tissue infections, along with elevated infective indices. The stable phase with an immune damage was defined as the initial clinical infective manifestation being controlled, but patients still having high levels of AIGAs and increased immune indices such as GLB, IgG, IgE, IgG4, eosinophils, and ESR; or high levels of AIGAs as well as clinical symptoms, such as nausea, anorexia, rash, and immune damage to the eyes, which are not caused by infection.

### Single-cell transcriptome sequencing

Raw sequencing data were processed with Illumina’s bcl2fastq and the 10x Genomics Cell Ranger pipeline (v3.1.0) for demultiplexing, alignment to the GRCh38/GRCm38 genome, and gene counting. This yielded 136,782 single cells from 3 healthy donors and 8 patients sequenced on the 10x Genomics Chromium platform.

Downstream analysis was performed in Seurat (v3.1.1). We filtered the data to retain 108,709 high-quality cells by removing cells with <500 or >5,000 genes, <500 UMIs, or >25% mitochondrial content, and genes detected in <3 cells. Data were log-normalized, and the top 10 principal components from PCA were used for SNN-based clustering and UMAP visualization. Cluster markers were identified using Seurat’s FindAllMarkers (Wilcoxon test), requiring expression in >10% of cells and an average log_2_FC > 0.25.

### Flow cytometry

Flow cytometry was performed to validate scRNA-seq findings. Lymphocyte subsets (T, NK, B, CD4^+^, CD8^+^) were quantified using the clinically approved BD Multitest™ 6-Color TBNK Reagent and analyzed in Kaluza software after FSC/SSC and CD45^+^ gating (**Supplementary Figure S1**).

T-helper subsets (Th1, Th2, Th17, Tc1, Tc2) were analyzed using a cytometric bead array (CBA) for IFN-γ^+^ (Th1/Tc1), IL-4^+^ (Th2/Tc2), and IL-17A^+^ (Th17) cells. Data were acquired on a calibrated flow cytometer and analyzed with FCAP Array v3.0 software, following quality control criteria of >1,800 total beads and ≥150 events per cytokine population.

B-cell subpopulations were characterized by multi-step FACS gating. After doublet exclusion and lymphocyte (FSC/SSC, CD45^+^) gating, total B cells were identified as CD19^+^. Subsets were defined using antibodies against CD27, IgD, IgM, CD38, CD24, and CD21 as: naïve (CD27^−^IgD^+^), marginal zone-like (CD27^+^IgD^+^), memory (CD27^+^CD38^dim^) including class-switched (IgD^−^IgM^−^) and unswitched (IgM^+^) variants, plasmablasts (CD27^hi^CD38^hi^), transitional (CD27^−^IgM^+^CD38^hi^CD24^+^), and CD21^low^ B cells (CD38^low^CD21^low^).

### Cytokine assay

Serum levels of the following cytokines were measured in 20 AIGAs-positive patients and 10 healthy controls: TNF-α (Cat. #m1064303V), GM-CSF (Cat. #m1025281V), IL-6 (Cat. #m1058097V), IL-5 (Cat. #m1058095V), IL-17 (Cat. #m1058051V), IL-4 (Cat. #m1058093V), TGF-β (Cat. #m1064258V), IL-12 (Cat. #m1058044), IL-2 (Cat. #m1058063V), and IFN-γ (Cat. #m1057856V). An indirect ELISA was performed according to the manufacturer’s instructions (Shanghai Enzyme-linked Biotechnology).

### Statistical analysis

The continuous data were presented as mean ± standard deviation (x ± s) for normally distributed data, and comparisons were made using independent samples t-tests. For non-normally distributed continuous data, the median and interquartile range were used, and comparisons were made using independent samples nonparametric rank sum tests. A significant difference was defined as a two-tailed *P* value < 0.05. Statistical analysis and graphing were performed using SPSS (version 27.0) and GraphPad Prism (version 10).

## Results

### Immune landscape of AIGAs immunodeficiency syndrome

This study enrolled 8 patients positive for AIGAs (case group), designated as P1 to P8, and 3 healthy controls (control group), designated as H1 to H3. The baseline characteristics of all participants are presented in **Supplementary Table 1**. There were no statistically significant differences in gender or age distribution between the case and control groups (*P* > 0.05).

**Figure 1:**
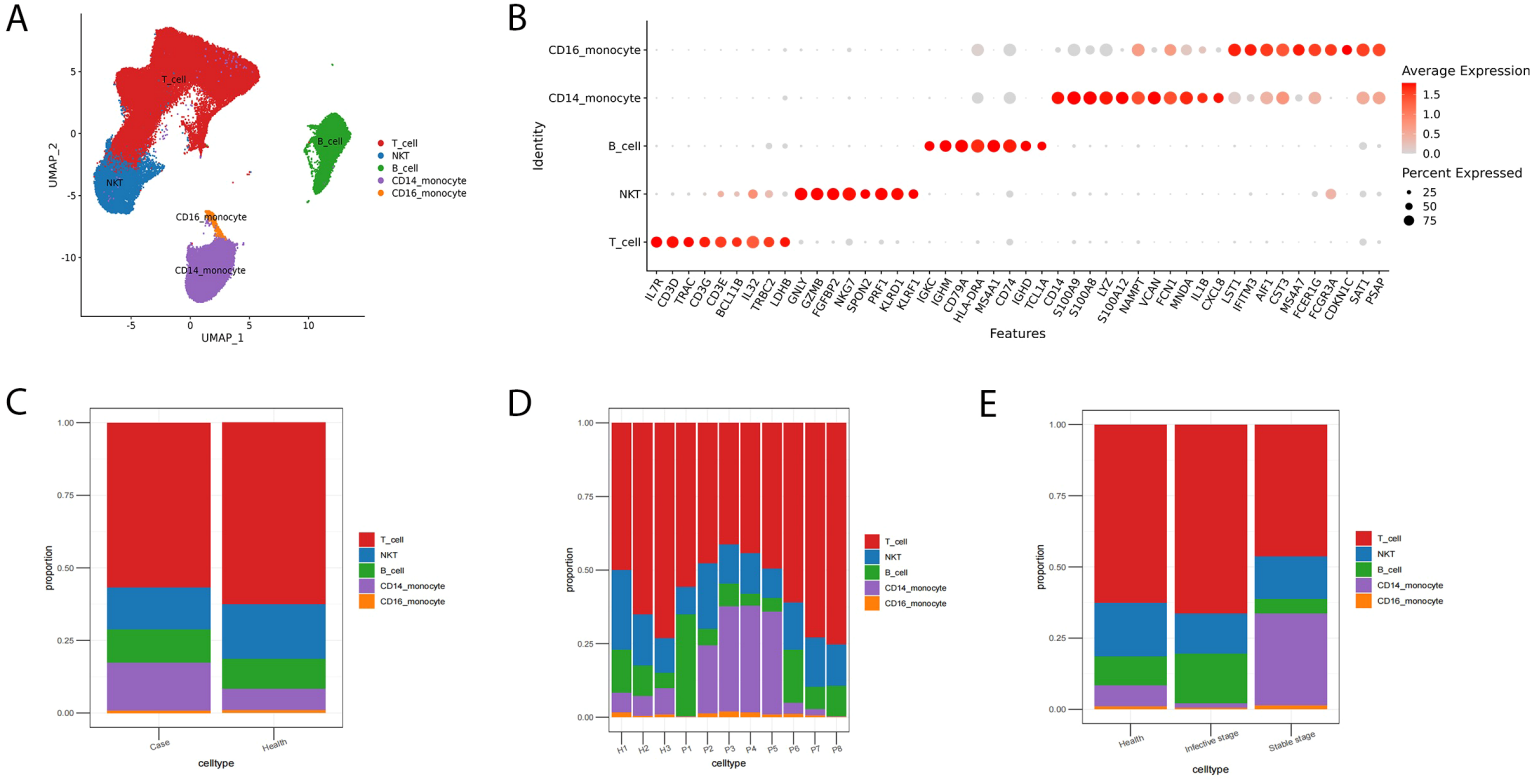
Immunological landscape of immunocytes from AIGAs immunodeficiency syndrome and the bar plot of the cell fractions of immunocyte subtypes in AIGAs-positive patients and the control group. **(A)** Two-dimensional UMAP visualization of 108709 cells from AIGAs-positive patients (P1–P8) and healthy controls (H1–H3). **(B)** Bubble plot showing the proportion (size) and relative expression levels (color) of marker genes in each immunocyte type. **(C)** Bar plot of immunocyte subtypes in the case and control groups. **(D)** Bar plot of immunocyte subtypes in all subjects. **(E)** Bar plot of immunocyte subtypes in AIGAs-positive patients at stable and infective phases.

After rigorous quality filtering, a total of 75,631 and 33,078 PBMCs were extracted from AIGAs-positive patients and healthy individuals, respectively. PBMCs immunocytes in AIGAs immunodeficiency syndrome mainly include T cells, B cells, monocytes, and natural killer T (NKT) cells. The immunological landscape of immunocytes was shown by uniform manifold approximation and projection (UMAP, **Figure 1A**). T cells specifically expressed *CD3D* and *CD3E*, which distinguished them from other clusters. B cells were characterized by high expression of *MS4A1* and *CD79A*, and NKT cells were identified based on the high expression of *NKG7*. Two subsets of monocytes were defined; that is, CD14^+^ monocytes had enriched *CD14*, *S100A8*, and *S100A9*, and CD16^+^ monocytes had enriched *FCGR3A* (*CD16*, **Figure 1B**).

The proportion of immunocytes differed significantly between AIGAs-positive patients and healthy controls. Specifically, patients exhibited a pronounced increase in CD14^+^ monocytes (16.35% vs. 7.42% in controls) and a moderate increase in B cells (11.6% vs. 10.24%). Conversely, we observed a decrease in T cells (56.78% vs. 62.56%) and NKT cells (14.43% vs. 18.86%) (**Figure 1C**).

The immunocyte proportion also varied among patients and across different disease phases in AIGAs-positive patients (**Figures 1D, E**). Compared with healthy individuals (Health), patients in the stable stage exhibited a marked increase in CD14^+^ monocytes (11.35% vs. 7.42%) alongside decreases in T cells (63.97% vs. 62.56%), NKT cells (13.98% vs. 18.86%), and B cells (10.04% vs. 10.24%). During the infective stage, this immune landscape shifted further: CD14^+^monocytes expanded dramatically to 22.52%, while T cells dropped substantially to 47.9%. Conversely, B cells increased to 13.51% compared to healthy (10.24%) group. NKT cells in the infective stage (14.97%) remained lower than healthy controls (18.86%).

### Activation of T cells in AIGAs immunodeficiency syndrome

**Figure 2:**
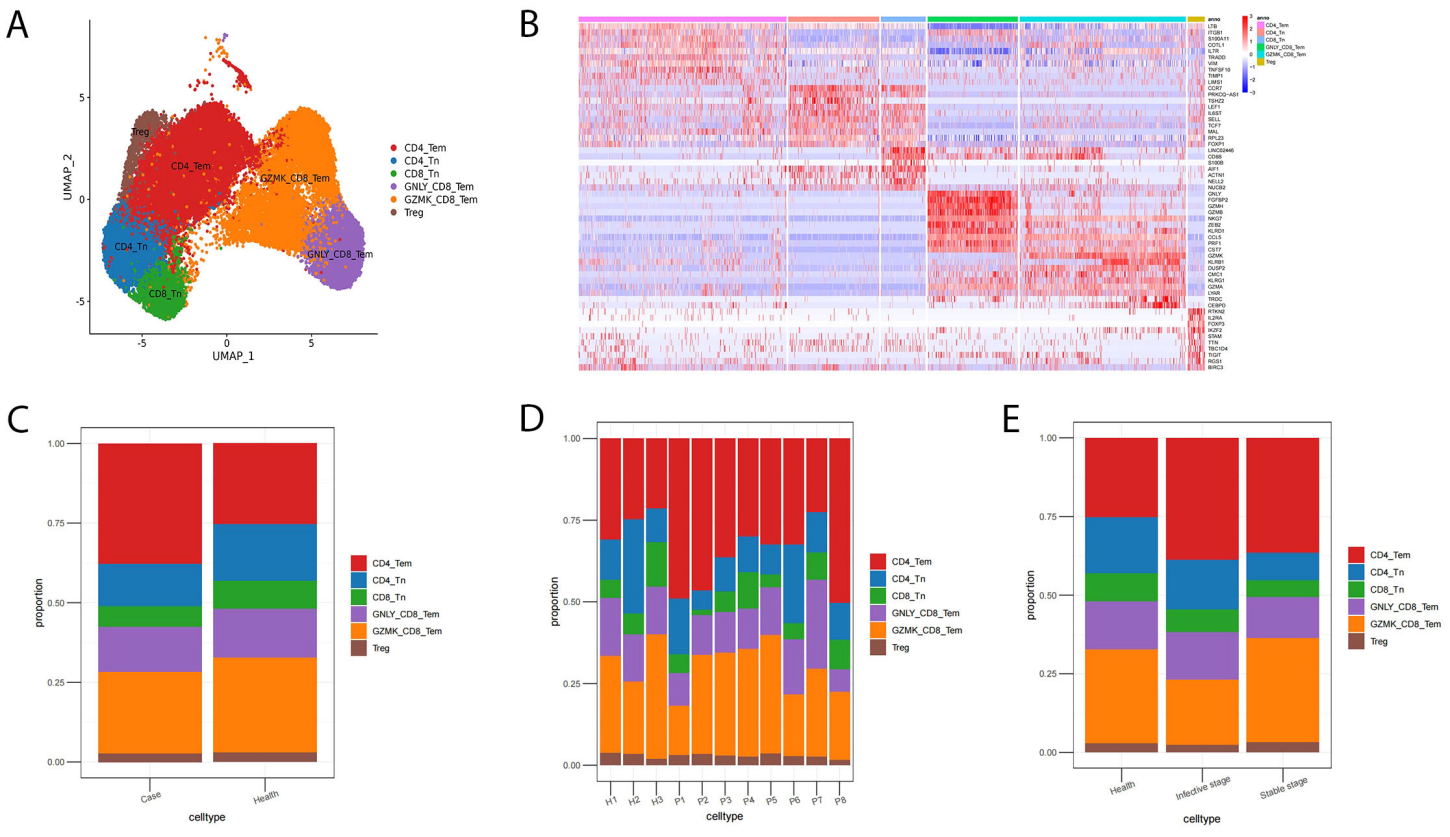
Landscape of T cell subsets in AIGAs-positive patients and the bar plot of cell fractions of T cell subtypes in AIGAs-positive patients and healthy controls. **(A)** Two-dimensional UMAP visualization of T cell subsets from AIGAs-positive patients (P1–P8) and healthy controls (H1–H3). **(B)** Heatmap showing the expression levels of selected gene markers for each T cell subsets. **(C)** Bar plot of T cell subtypes in the case and control groups. **(D)** Bar plot of T cell subtypes in all subjects. **(E)** Bar plot of T cell subtypes in AIGAs-positive patients at infective and stable phases.

T cells were categorized into two groups, namely, CD8^+^ T and CD4^+^ T cells, based on the expression of *CD4*, *CD8A*, and *CD8B*. The immunological landscape of T cell subsets was visualized using uniform manifold approximation and projection (UMAP) in **Figure 2A**. **Figure 2B** presents a heatmap of the expression levels of specific gene markers in each T cell subset. An increase in the number of CD4^+^ T cells (51.78%) and a decrease in the level of CD8^+^ T cells (44.53%) were observed in the case group (**Figure 2C**). The proportion of T cell subsets varied between individual patients and patients under different conditions (**Figures 2D, E**).

Naive CD8^+^ T cells (CD8_Tn; *LEF1*, *SELL*, *TCF7*), GNLY effector memory CD8^+^ T cells (GNLY_CD8_Tem; *GNLY*, *GZMH*, *GZMB*), and GZMK effector memory CD8^+^ T cells (GZMK_CD8_Tem; *GZMK*, *GZMA*) were identified in the CD8^+^ T cell population (17). CD4^+^ T cells mainly consist of effector memory CD4^+^ T cells (CD4_Tem; *LTB*, *IL7R*), naive CD4^+^ T cells (CD4_Tn; *LEF1*, *SELL*, *TCF7*), and regulatory cells (Treg; *FOXP3*).

**Figure 3:**
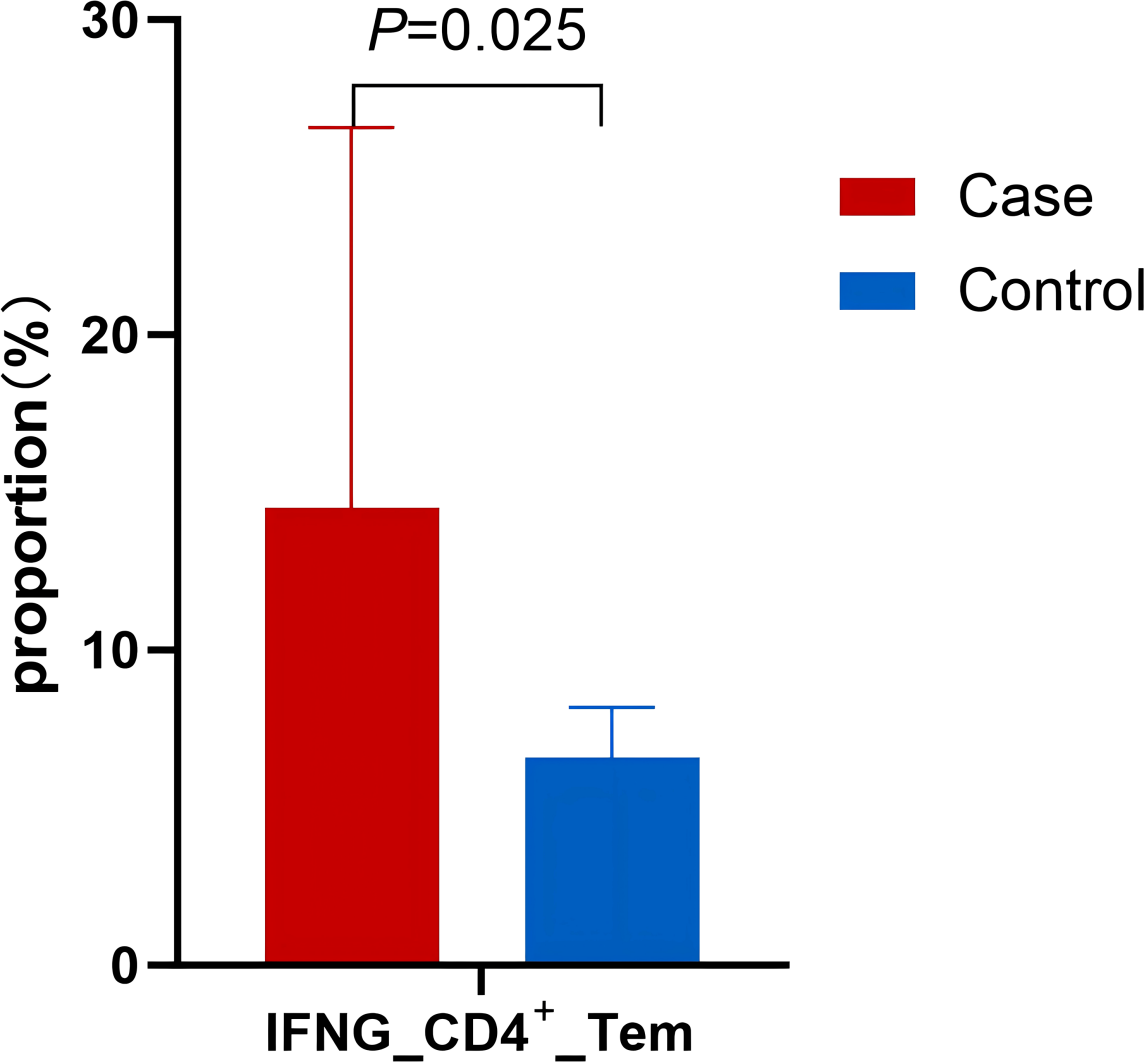
Proportion of IFNG_CD4_Tem and Th1 (IFN-γ^+^CD4^+^) between AIGAs-positive patients (case) and control.

The level of CD4_Tem increased in the case group, and that of CD4_Tn decreased. Upon further analysis, CD4_Tem showed a higher expression of the IFN-γ gene (*IFNG*) in the case group (16.62%) than in the control group (6.94%) (**Figure 3**).

### Increased B cells expressing IFN-stimulated genes and decreased plasma in AIGAs immunodeficiency syndrome

**Figure 4:**
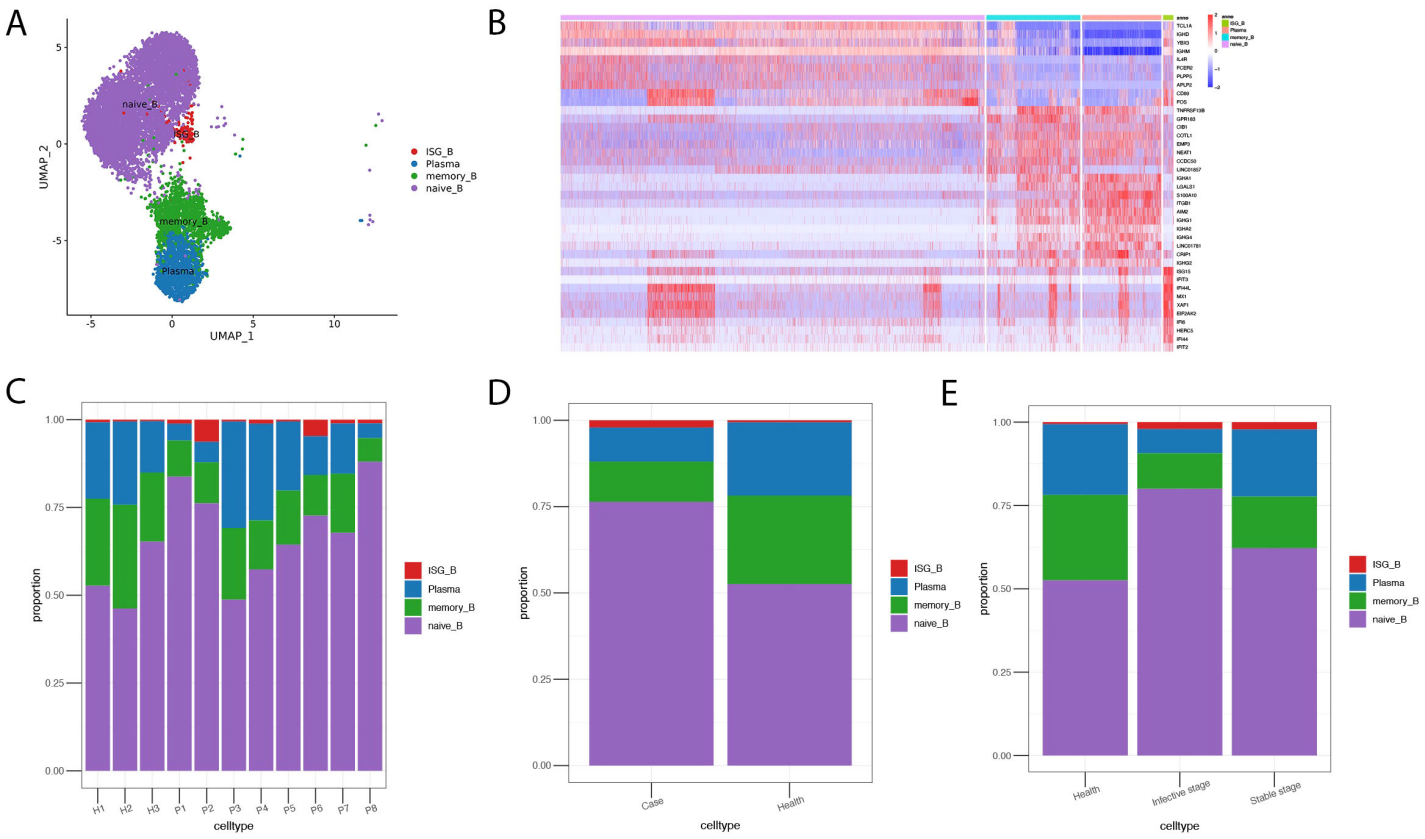
Landscape of B cell subsets in AIGAs-positive patients and the bar plot of cell fractions of B cell subtypes in AIGAs-positive patients and healthy controls. **(A)** Two-dimensional UMAP visualization of B cell subsets from AIGA-positive patients (P1–P8) and healthy controls (H1–H3). **(B)** Heatmap showing the expression levels of the selected gene markers of each B cell subset. **(C)** Bar plot of B cell subsets in all subjects. **(D)** Bar plot of B cell subsets in the case and control groups. **(E)** Bar plot of B cell subsets in AIGA-positive patients at infective and stable phases.

The B cells subgroup consists of naive B cells expressing *IGHD*, *IGHM*, and *TCL1A* (naive_B), memory B cells expressing *AIM2* and *TNFRSF13B* (memory_B), plasma cells expressing *IGHA1*and *IGHG1* (plasma), and an atypical B cell subpopulation expressing IFN-stimulated genes (ISG) (ISG_B) (**Figures 4A, B**). The proportions of these B cell subpopulations varied among individuals (**Figure 4C**). B cell subpopulation analysis revealed a profound shift in AIGAs-positive patients, characterized by an expansion of naive B cells (78.21% vs. 54.32% in health) and ISG^+^ B cells (2.95% vs. 0.53%), alongside a contraction of memory B cells (9.54% vs. 27.35%) and plasma cells (9.30% vs. 17.79%). This distribution was maintained across disease stages, with ISG^+^ B cells persistently elevated (stable: 4.03%; infective: 2.70%) and memory B cells remaining suppressed (stable: 14.35%; infective: 8.41%). Notably, plasma cells were decreased in both the stable and infective stages (21.57% and 6.44%, respectively) compared to health (17.79%) (**Figures 4D, E**).

### Differentially expressed genes analysis in AIGAs immunodeficiency syndrome

**Figure 5:**
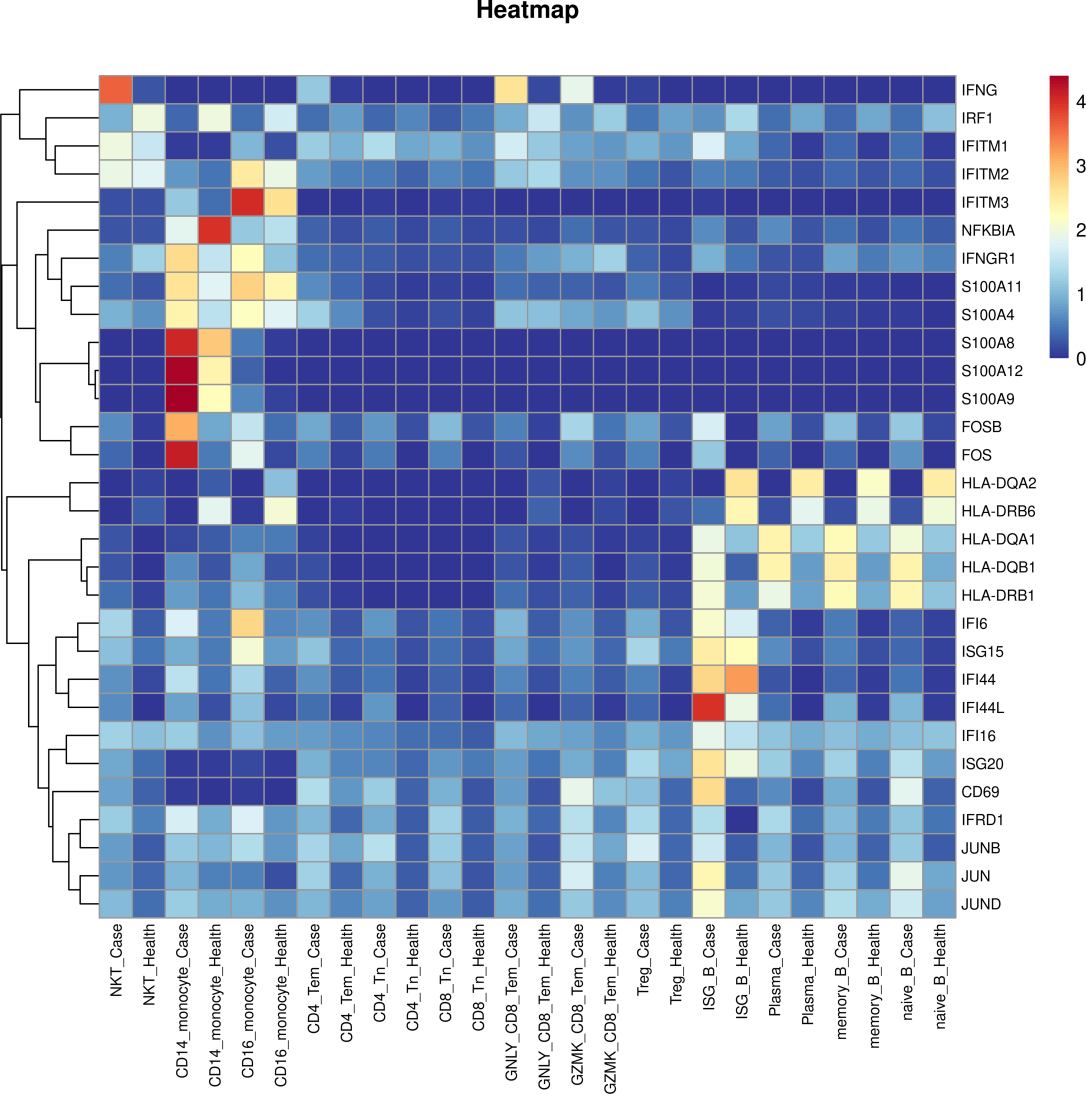
Heatmap showing expression of upregulated and downregulated genes in patients with AIGAs immunodeficiency syndrome. Rows represent each gene in each cell subgroup, and columns represent each cell subgroup between cases and controls. The color of each square denotes the level of gene expression, with different colors indicating upregulation or downregulation.

Differentially expressed genes for each cell subsets were analyzed using the likelihood-ratio test. Upregulated genes were selected based on both a log2 FC ≥ 0.36 and a *P* value ≤ 0.01 in AIGAs immunodeficiency syndrome patients. Analysis of T cell subsets revealed a significant upregulation of interferon response genes, including *IFNG*, *IFRD1*, *ISG15*, *ISG20*, *IFITM2*, *IFI6*, and *IFI44L*, in most T cell subtypes (**Figure 5**). *HLA-DRB1*, *HLA-DQB1*, and *HLA-DQA1*, were also upregulated in most T cell subtypes (**Figure 5**). Genes belonging to the Fos gene family, such as *FOS* and *FOSB*, along with those from the JUN family, including *JUN*, *JUNB*, and *JUND*, were broadly expressed in all T cell subtypes (**Figure 5**).

Subsequently, the DEGs of B cell subsets were analyzed. *IFI44L*, *IFITM1*, *HLA-DRB1*, *HLA-DQB1*, *HLA-DQA1*, *JUND*, *JUNB*, *JUN*, *FOSB*, *FOS*, and *CD69* were significantly upregulated in most B cell subtypes (**Figure 5**). *IGHE* was significantly upregulated in plasma cells in patients (**Supplementary Figure S2**).

Monocyte upregulated differential gene analysis also revealed a series of interferon response genes, including *IFITM3*, *IFI6*, *IFI44L*, *IFI44*, *IFI16*, *IFNGR1*, *IFITM2*, *IFRD1*, and *ISG15* genes. Genes such as *HLA-DRB1*, *HLA-DQB1*, *HLA-DQA1*, *JUND*, *JUNB*, *JUN*, *FOSB*, and *FOS*, which are upregulated in most subtypes of T and B cells, were also highly expressed in monocytes. Additionally, *S100A9*, *S100A12*, *S100A4*, *S100A11*, and *S100A8* were highly upregulated in monocytes (**Figure 5**).

Moreover, the upregulated differential genes of NKT cells were simultaneously analyzed, and a series of interferon-stimulated genes were found, including *IFNG*, *IFI44L*, *IFI6*, *ISG15*, *ISG20*. The expression of *HLA-DRB1*, *HLA-DQB1*, and *HLA-DQA1* was also upregulated (**Figure 5**).

The analysis of the up-regulated genes in the above cell subsets revealed that a number of interferon genes and human leukocyte antigen genes, as well as the *FOS* and *JUN* gene families, were up-regulated in most cell subsets of the patients (**Figure 5**).

Finally, we analyzed downregulated differentially expressed genes for each cell subset and chose downregulated genes based on log2 FC ≤ 0.36 and P value ≤ 0.01 in AIGAs immunodeficiency syndrome patients. *IRF1* genes were downregulated in all cell subgroups, while *HLA-DQA2* and *HLA-DRB6* were downregulated in most cell subgroups (**Figure 5**). *IGHA1* and *IGHA2* genes was significantly downregulated in plasma cells (**Supplementary Figure S2**). The complete datasets corresponding to all these analyses are provided in **Supplementary Table 2**.

### Disease-associated pathways revealed in patients with AIGAs immunodeficiency syndrome

**Figure 6:**
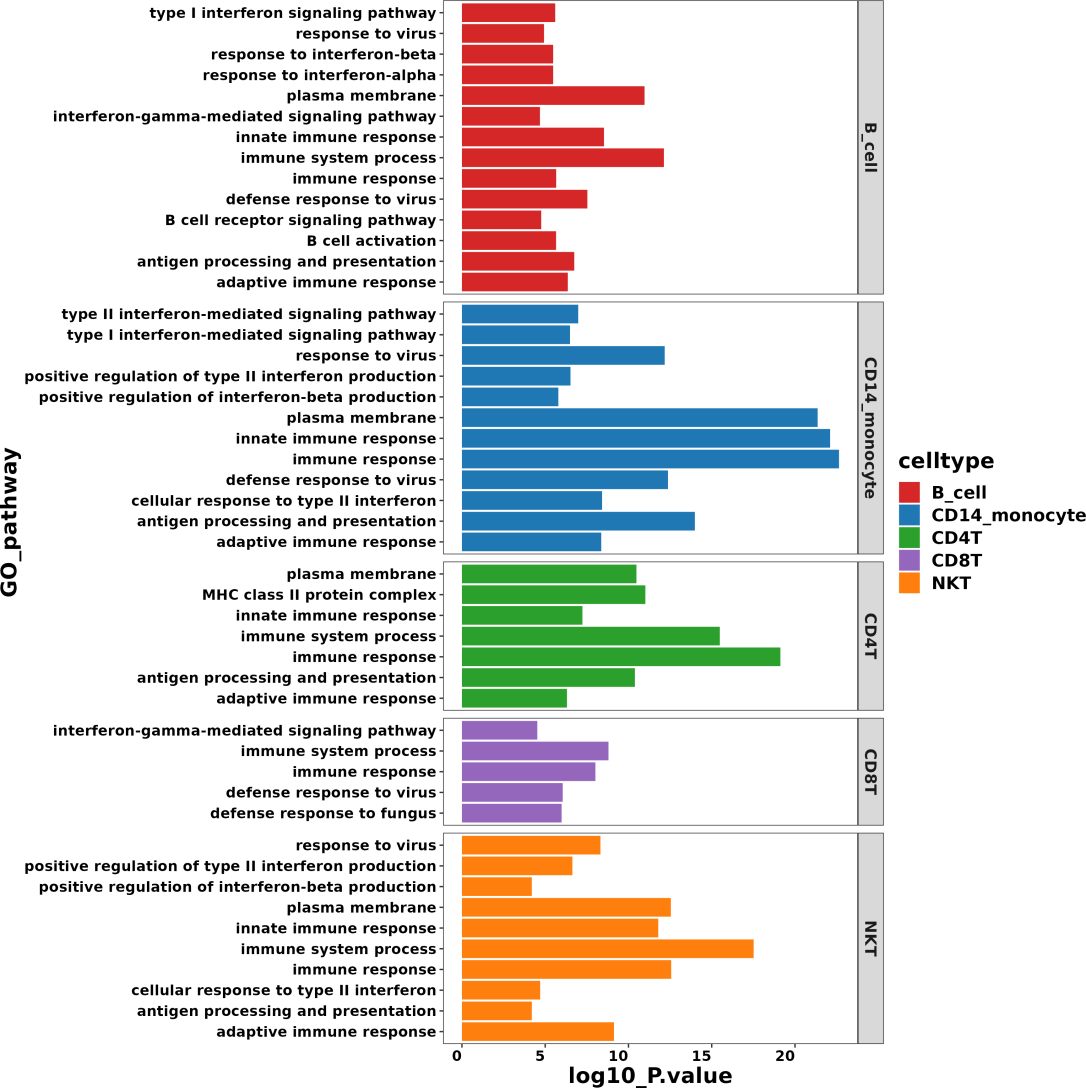
GO pathway enrichment analysis of the differentially expressed genes in patients with AIGAs syndrome.

Gene Ontology (GO) and the Kyoto Encyclopedia of Genes and Genomes (KEGG) pathway analyses were utilized to investigate cell subset-specific pathways in patients with differentially expressed genes (DEGs). GO analysis revealed that the majority of the gene products were located in the plasma membrane, suggesting a focus on cell-surface communication and environmental sensing. The analysis also indicated that the metabolic pathways of multiple cell subsets were associated with immune processes or immune responses. Notably, interferon signaling, including type I IFN (mainly IFN-α and IFN-β) and type II IFN, was activated in B cells, CD14+ monocytes, and NKT cells (**Figure 6**). This broad activation of IFN pathways across distinct immune lineages implies a robust, systemic antiviral state or a heightened inflammatory environment in the patients. Furthermore, enrichment was observed in pathways related to defense responses to viruses and fungi, antigen processing and presentation, as well as B cell activation (**Figure 6**). The co-enrichment of antiviral defense and antigen presentation pathways highlights a coordinated innate and adaptive immune mobilization, while enhanced B cell activation signals suggest an ongoing humoral immune response.

**Figure 7:**
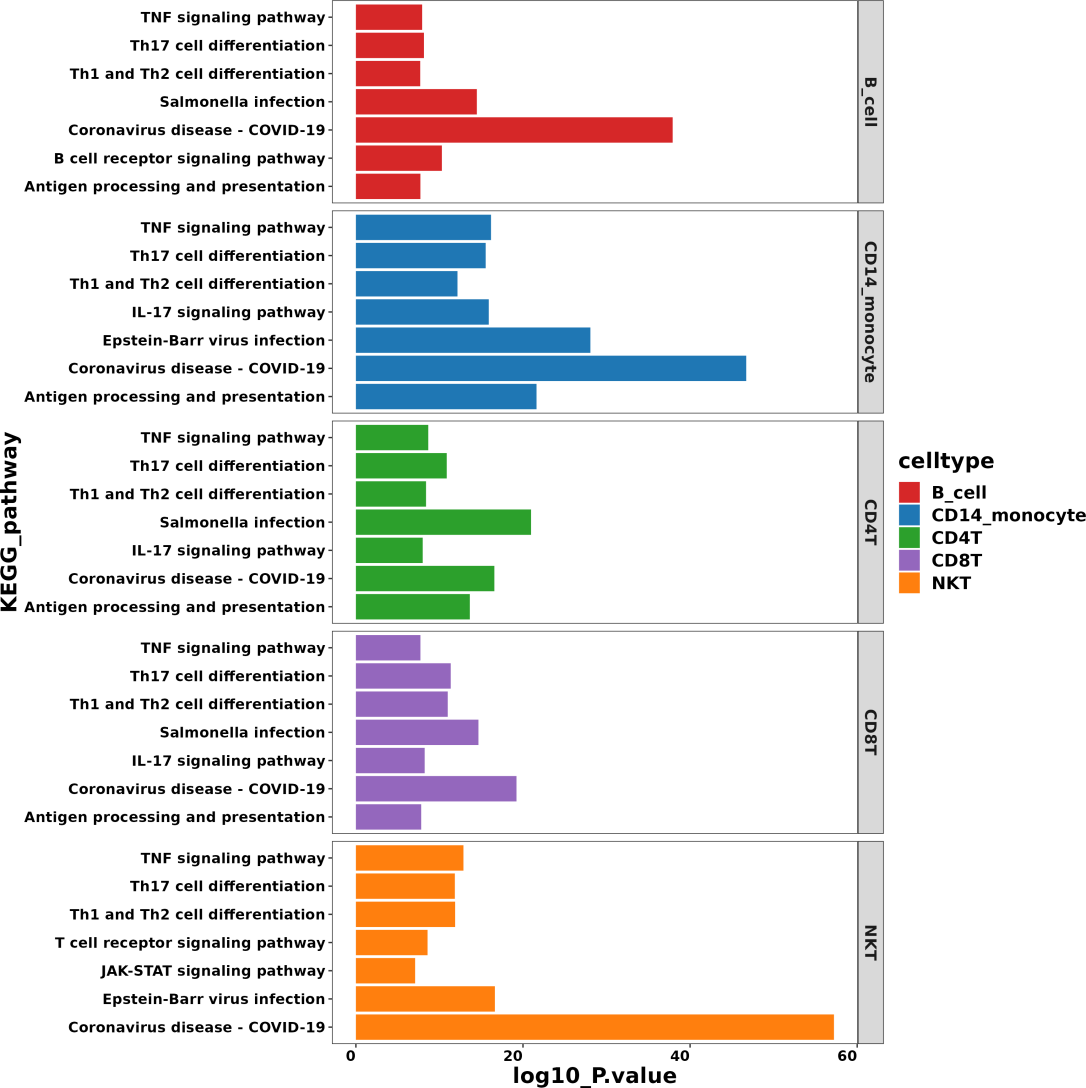
KEGG pathway enrichment analysis of the differentially expressed genes in patients with AIGAs syndrome.

The KEGG pathway analysis was simultaneously conducted, revealing enrichment of several immune-associated pathways across various cell subsets. These pathways include Antigen Processing and Presentation, Th17 Cell Differentiation, Th1 and Th2 Cell Differentiation, and the IL-17 Signaling Pathway. The enrichment of these T cell polarization and IL-17 pathways points to a complex T-cell-mediated immune landscape, potentially involving both pro-inflammatory (Th1/Th17) and other adaptive mechanisms. Additionally, pathways related to infectious diseases such as Coronavirus Disease (COVID-19), Epstein-Barr Virus Infection, and Salmonella Infection were identified (**Figure 7**). This pattern suggests that the patients’ immune gene expression profile shares similarities with responses to specific viral and bacterial pathogens, which could provide clues about the disease etiology or triggering factors. Moreover, signal transduction pathways such as the TNF Signaling Pathway and the JAK-STAT Signaling Pathway were enriched as well (**Figure 7**). The enrichment of these key pro-inflammatory (TNF) and cytokine signaling (JAK-STAT) hubs further underscores the presence of a strong inflammatory milieu and offers potential mechanistic insights and therapeutic targets.

### Cell-cell communication uncovers Th1/monocyte imbalance in AIGAs immunodeficiency syndrome

**Figure 8:**
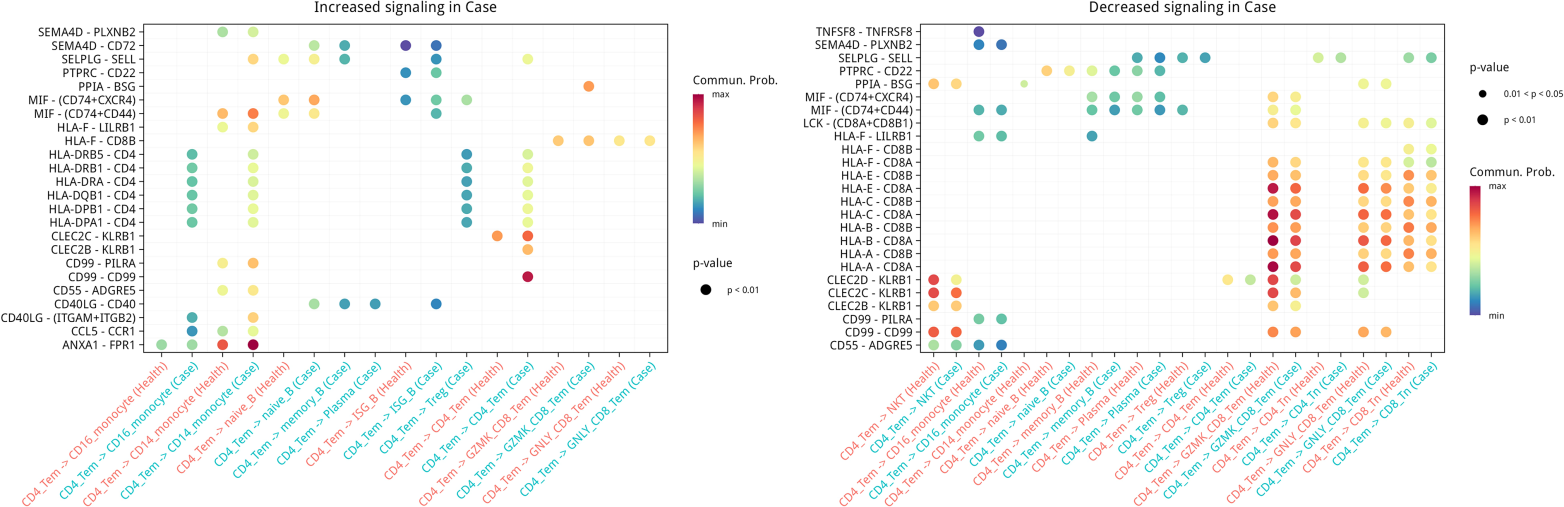
Bubble-map of inter-cellular communication inferred for CD14^+^monocytes..

**Figure 9:**
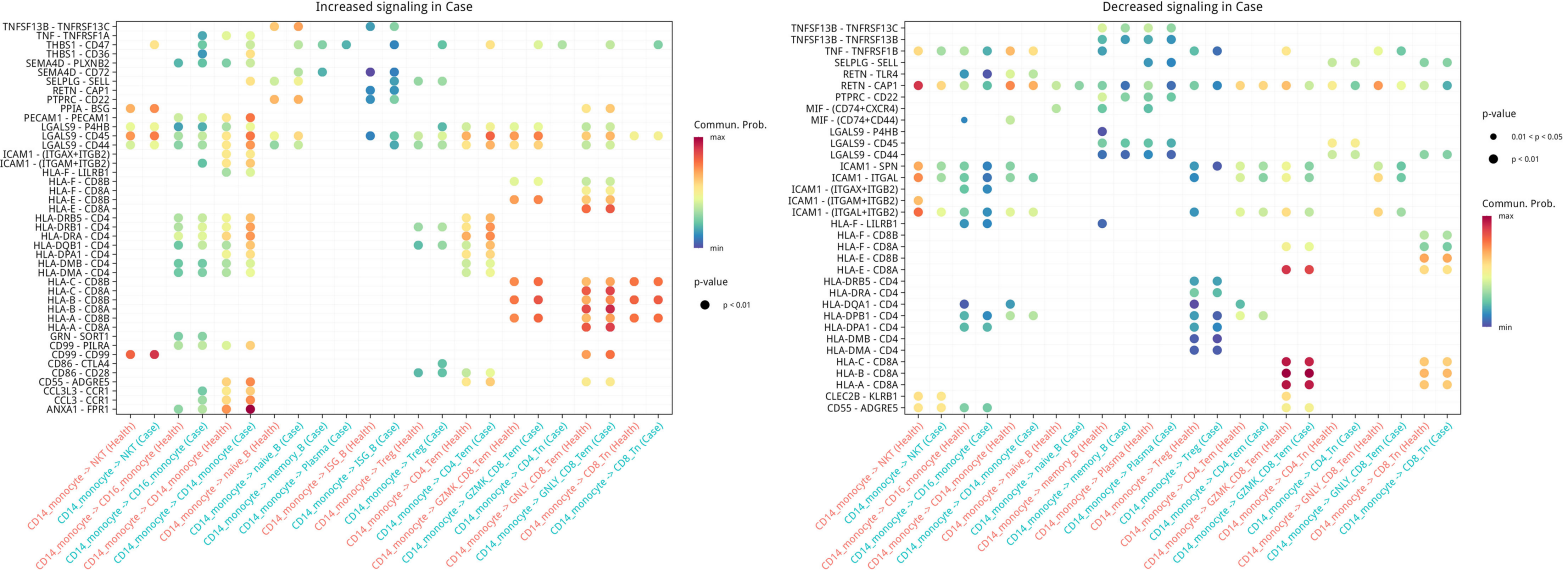
Bubble-map of inter-cellular communication inferred for CD4_Tem cells.

Given the marked increase of CD4_Tem and CD14^+^ monocytes in AIGAs immunodeficiency syndrome patients, we specifically investigated their intercellular communication networks. CellChat analysis disclosed a bidirectional augmentation of both pro-inflammatory and antigen-presenting pathways in AIGAs peripheral blood (**Figures 8**, **9**).

Key upregulated ligand-receptor axes included: the adhesion/recruitment pair SELPLG-SELL, the inflammatory amplifier MIF-(CD74+CD44), MHC-II antigen presentation (e.g., HLA-DRB5/DRB1/DRA-CD4), and the potential negative feedback mediator HLA-F-LILRB1. Notably, the anti-inflammatory resolution axis ANXA1-FPR1 was also prominently active, indicating an insufficient compensatory response against the overall inflammatory milieu. These results collectively demonstrate, at single-cell resolution, a communication imbalance in AIGAs peripheral blood defined by heightened antigen presentation and leukocyte recruitment alongside overwhelmed inhibitory signaling, thereby elucidating potential mechanisms underlying sustained Th1 inflammation and monocyte hyperactivation.

### Pseudotime mapping reveals aberrant T/B differentiation in AIGAs immunodeficiency syndrome

Pseudotemporal ordering, seeded by CD4_Tn and CD8_Tn and sequentially resolved to CD4_Tem and GNLY^+^CD8_Tem, corroborated the fidelity of T-cell sub-cluster annotation (**Supplementary Figure S3**).

Compared with healthy controls, AIGAs-derived trajectories were globally shifted toward terminal effector fates; notably, the second bifurcation was significantly skewed toward CD4_Tem commitment, indicating that peripheral T cells in patients persist in a “high-effector, low-quiescent” state and providing further evidence of disease-associated hyper-immunity. Guided by this observation, we focused on CD4^+^ T-cell subsets. Lineage-restricted pseudotime (**Supplementary Figure S4**) bifurcated from CD4_Tn into CD4_Tem and Treg branches, recapitulating a “naive→ effector/regulatory” dichotomy. Cell density at pseudotime node 4 was markedly increased in patients and preferentially routed to CD4_Tem. Differential gene analysis of patient-versus control-derived CD4_Tem at this node identified significant up-regulation of LYZ, S100A8 and S100A9—perfectly mirroring the global differential signature(**Supplementary Figure S5**).

**Figure 10:**
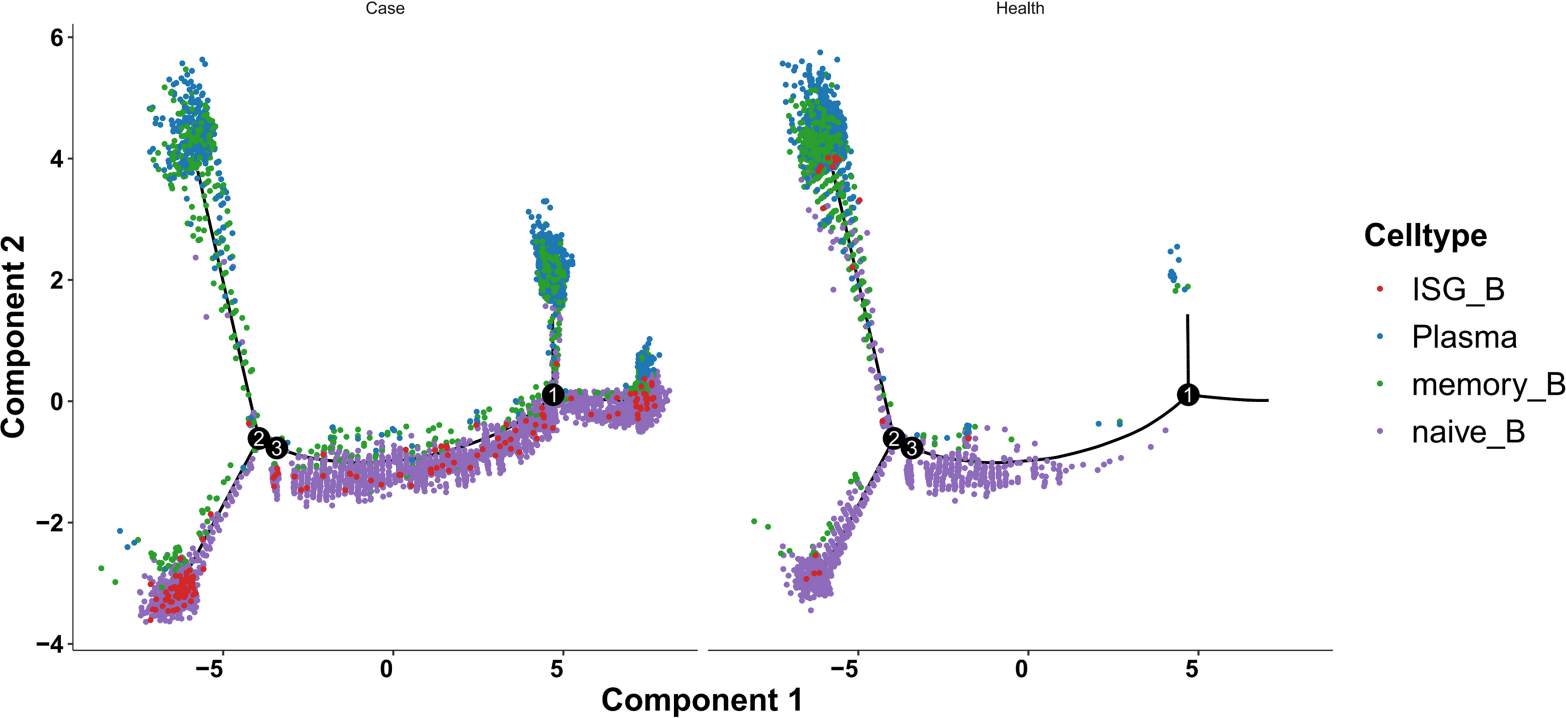
Monocle 2 pseudotime trajectory of B cells, coloured by Seurat-annotated subtypes, comparing case and control samples.

**Figure 11:**
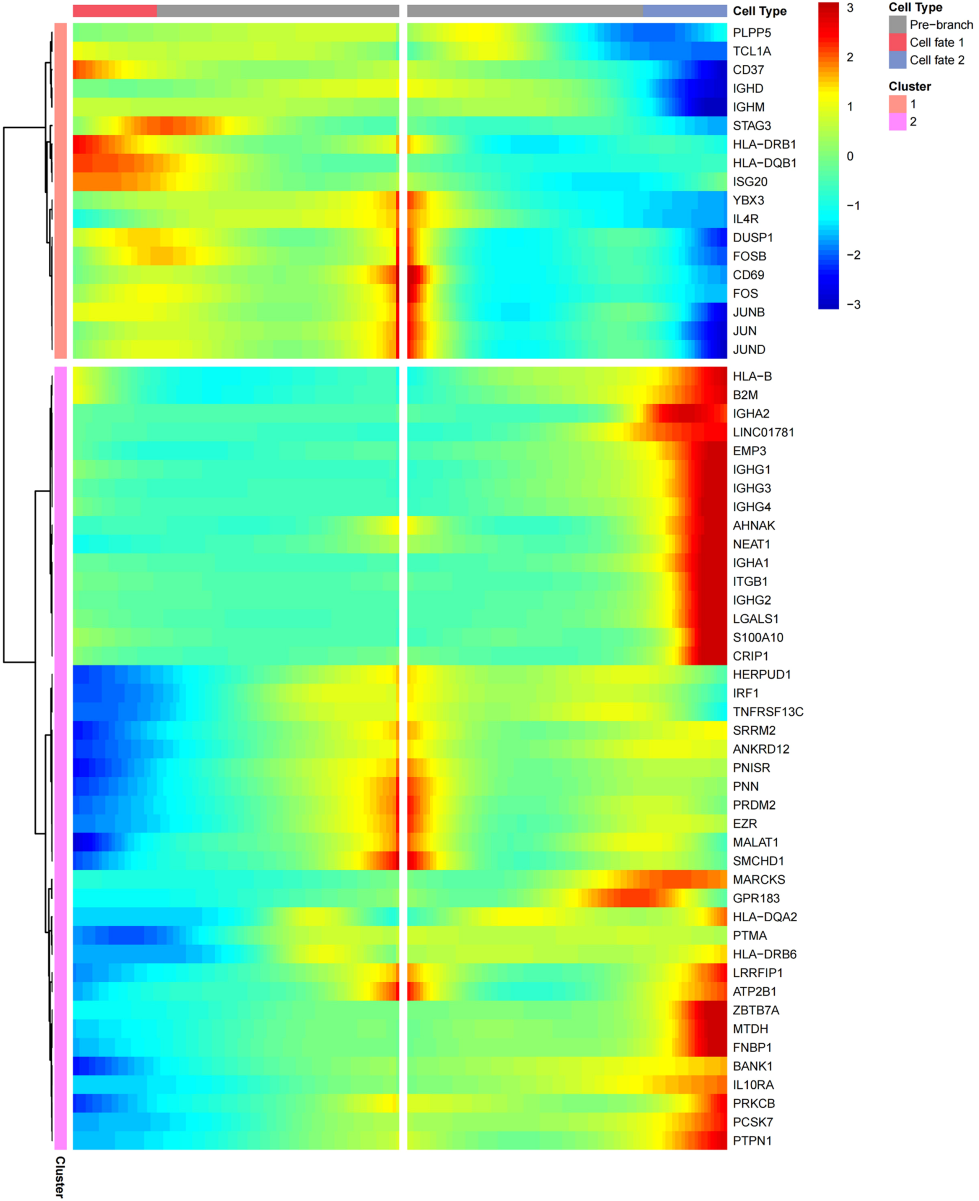
Pseudotime-resolved expression heat-map of genes that vary significantly along the Monocle 2 trajectory in B-cell node 2.

**Figure 12:**
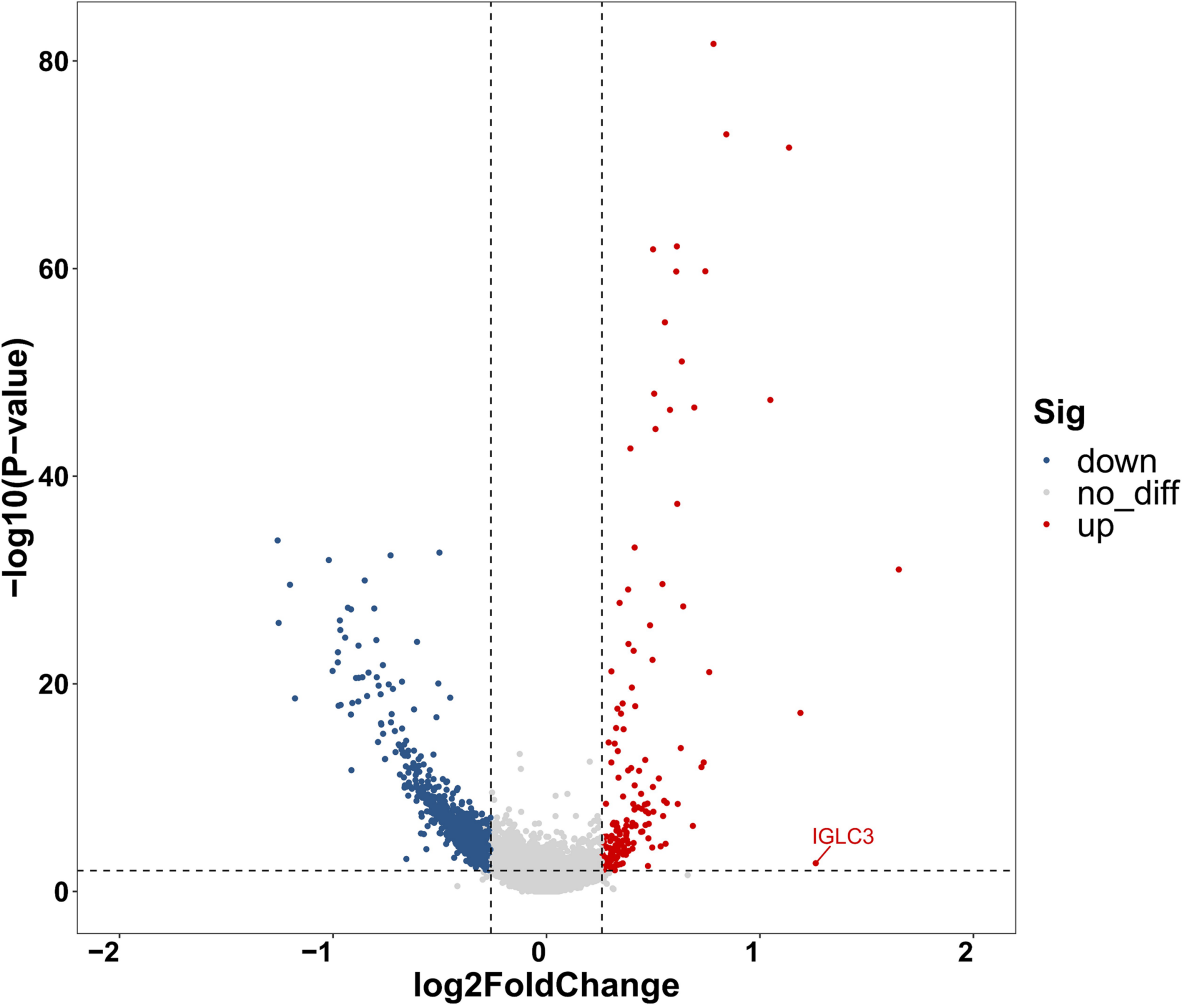
Volcano plot comparing B-cell node 1 versus node 2 (patient group).

B-cell pseudotime reconstruction revealed that, although both groups displayed the canonical naive→memory→plasma-cell axis, patients exhibited a substantially more intricate differentiation topology (**Supplementary Figure S6**). Three disease-specific features were evident: (i) an ISG_B-cell cluster emerged exclusively in patients, implying aberrant interferon-pathway activation within the patient-specific trajectory (**Figure 10**); (ii) branch-point analysis showed an extra bifurcation (node 1) in patients that generated plasma-cell and memory-B-cell subsets completely absent in controls, whereas both groups shared these populations at node 2 (**Figure 10**); and (iii) BEAM analysis of node 2 demonstrated that the earlier-emerging plasma cells were enriched for *IGHG1*, *IGHA2*, *IGHG3* and *IGHG4* (**Figure 11**). Consequently, we performed differential gene analysis on node 2 and node 1 from the patient group. This analysis revealed that at node 2, the later-emerging plasma cell fraction displayed selective up-regulation of *IGLC3* (**Figure 12**).

### Flow cytometry showed increased Th1 cells in AIGAs immunodeficiency syndrome

**Figure 13:**
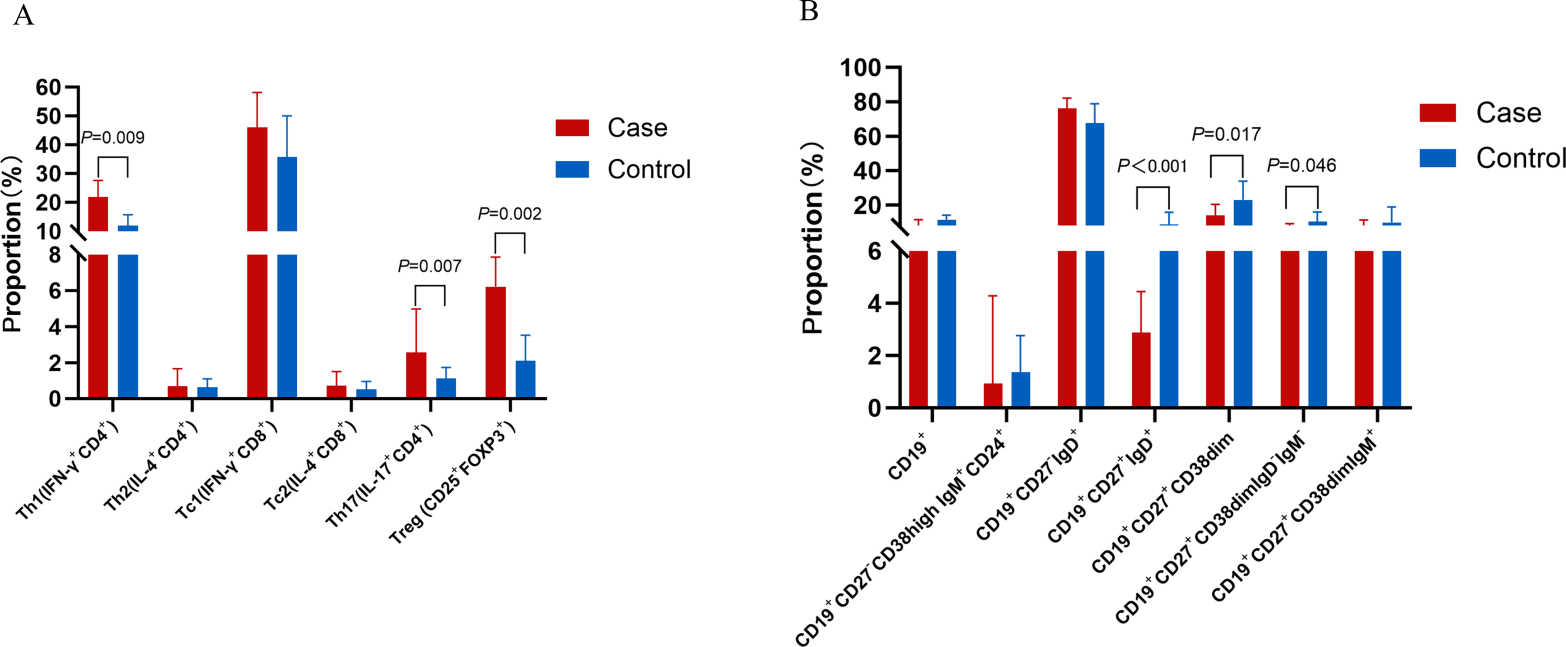
The proportion of T and B lymphocyte subsets in AIGAs-positive patients and healthy controls. **(A)** The proportion of T lymphocyte subsets. **(B)** The proportion of B lymphocyte subsets (CD19+: Total B cells; CD19+CD27-CD38high IgM^+^CD24^+^: Transitional B cells; CD19^+^CD27-IgD+: Naive B cells; CD19+CD27^+^IgD+: Marginal zone B cells; CD19^+^CD27^+^CD38dim: Memory B cells; CD19^+^CD27^+^CD38dimIgD-IgM-: Classical immunoglobulin class-switched B cells; CD19+CD27+CD38dimIgM+: non-immunoglobulin class-switched B cells).

A total of 25 individuals were enrolled, including 15 AIGAs-positive patients and 10 healthy controls. As shown in and **Figure 13A**, the proportion of Th1(IFN-γ^+^CD4^+^) cells(21.84[14.87-27.57] vs.11.96[7.19-15.74] in control), Th17(IL-17^+^CD4^+^) cells(2.56[1.58-4.98] vs. 1.13[0.86-1.74]), Treg (CD25^+^FOXP3^+^)(6.22[3.51-7.86] vs.2.11[1.54-3.53]) in CD4^+^ T cells increased in AIGAs-positive patients (*P* < 0.05). Conversely, no statistically significant variances were observed in the distribution of Th2(IL-4^+^CD4^+^), Tc1(IFN-γ^+^CD8^+^), and Tc2(IL-4^+^CD8^+^) between the two groups (*P*>0.05).

Subsequently, B cell subsets were evaluated. Compared to healthy controls, AIGAs-positive patients exhibited a significant reduction in the frequencies of marginal zone B cells (CD19^+^CD27^+^IgD^+^; 2.87% [IQR 1.71-4.45] vs. 8.60% [IQR 6.77-15.65]), memory B cells (CD19^+^CD27^+^CD38^dim^; 13.85% [5.72-20.23] vs. 22.96% [16.39-33.83]), and class-switched memory B cells (CD19^+^CD27^+^IgD^−^IgM^−^; 6.11% [2.39-9.10] vs. 10.18% [5.35-15.77]) (**Figure 13B**).

### The level of IFN-γ in patients with AIGAs immunodeficiency syndrome was not decreased

In our study, we assessed the serum levels of TNF-α, GM-CSF, IL-6, IL-5, IL-17, IL-4, TGF-β, IL-12, IL-2, and IFN-γ in 20 AIGAs-positive patients and 10 healthy controls. As shown in **Table 1**, there were no significant differences in IFN-γ, IL-2, TNF-α, IL-12, IL-4, IL-6, and TGF-β levels between the two groups (*P* = 0.008). However, AIGAs-positive patients had significantly lower levels of IL-5 (*P* = 0.009) and IL-17 (*P* = 0.044), while GM-CSF levels were higher (*P* = 0.002) compared to healthy controls.

**Table 1:** Comparison of serum cytokines between AIGAs-positive patients and healthy controls.

Cytokines	Case (n=20)	Control (n=10)	*P* value
IFN-γ (pg/ml)	302.27[206.40-505.18]	254.91[190.00-315.25]	0.286
IL-2 (pg/ml)	221.31[109.52-398.16]	143.03[45.88-181.61]	0.082
TNF-α (pg/ml)	82.25±39.88	67.33±27.01	0.307
IL-12 (pg/ml)	9.75[3.24-17.04]	5.07[2.79-6.70]	0.307
IL-4 (pg/ml)	61.74[43.59-102.42]	51.78[51.14-60.09]	0.746
IL-5 (pg/ml)	12.96[12.05-17.99]	19.93[17.94-31.83]	0.008
IL-6 (pg/ml)	32.39±9.37	39.80±7.82	0.055
IL-17 (pg/ml)	357.69±74.50	443.48±86.43	0.009
TGF-β (ng/ml)	61.94[43.59-102.42]	82.89[45.10-90.55]	0.812
GM-CSF (pg/ml)	879.49[706.10-1084.80]	577.65[518.92-677.50]	0.002

## Discussion

This study presents the first single-cell RNA sequencing of PBMCs from patients with AIGAs immunodeficiency syndrome, revealing a complex and heterogeneous immune landscape dominated by T cells, B cells, monocytes, and NKT cells. We identified prominent expansions of CD4^+^ Tem cells and CD14^+^ monocytes, accompanied by Th1 elevation and increased IFN-γ levels. In the B-cell compartment, increases in ISG^+^ and naive B cells were observed alongside reductions in plasma and memory B cells. CellChat analysis demonstrated that reinforced crosstalk between CD4^+^ Tem and CD14^+^ monocytes—through enhanced antigen presentation, inflammatory signaling, and leukocyte recruitment—drives Th1 polarization and monocyte hyperactivation, consistent with elevated serum IFN-γ. Pseudotime analysis further uncovered a skewed T-cell differentiation toward terminal effectors (enriched for LYZ and S100A8/A9) and aberrant B-cell development featuring an emergent ISG^+^ subpopulation, premature plasma cell differentiation, and IGLC3-biased class switching. Collectively, these findings establish AIGAs immunodeficiency syndrome as a polygenic disorder involving interferon and HLA-related genes, delineating its distinctive immunopathology and providing novel mechanistic insights into its pathogenesis.

During the infectious phase of AIGAs immunodeficiency syndrome, T cell levels—particularly CD4^+^ Tem cells—were elevated. Although total T cells declined in the stable phase with immune damage, CD4^+^ Tem levels remained higher than in healthy controls. Further analysis showed an increase in IFNG-expressing CD4^+^ Tem cells, consistent with flow cytometry data revealing more Th1 cells. ELISA measurements indicated elevated IFN-γ in patients, though not statistically significant, suggesting intact T cell activation and IFN-γ production, yet functional neutralization by AIGAs. Intracellular pathogens likely drive Th1 differentiation (18), explaining the Th1 increase in AIGAs. Persistent IFN-γ secretion from these cells elevates IFN-γ, IL-2, and TNF-α levels. As infection stabilizes, Th1 differentiation declines, reducing T cell counts. We speculate that sustained AIGAs production may result from IFN-γ itself acting as an antigen, promoted by aberrant antigen presentation.

Pseudotime analysis revealed a skewed T-cell differentiation toward terminal effector phenotypes in AIGAs immunodeficiency syndrome, accompanied by upregulation of LYZ and S100A8/A9. As classic myeloid alarmins, S100A8/A9 are typically restricted to monocytes/macrophages and neutrophils (19) and serve as inflammatory markers in conditions such as rheumatoid arthritis (20). Their aberrant expression in T cells suggests cellular identity blurring and functional adaptation under intense inflammatory pressure, potentially amplifying overall immune dysregulation. Cell communication analysis further uncovered a pro-inflammatory circuit between CD14^+^ monocytes and CD4^+^ Tem cells: monocytes persistently activate T cells via heightened antigen presentation (MHC–II–CD4); the MIF–(CD74+CD44) axis amplifies inflammatory cascades, likely inducing TNF-α and IL-1β production; and SELPLG–SELL-mediated recruitment enriches immune cells at inflammatory sites. This circuit correlates with elevated serum IFN-γ and Th1 polarization, potentially driving chronicity and tissue injury in AIGAs immunodeficiency syndrome.

B cell analysis revealed phase-dependent fluctuations in abundance, with elevations during infection and reductions in stable disease, reflecting dynamic immune adaptation. While total B cells increased during infection—consistent with expected antigen-driven expansion—plasma cell numbers were reduced in both phases, falling by 11% in AIGAs-positive patients. Flow cytometry confirmed a specific decrease in class-switched, antibody-secreting B cells. Pseudotime analysis resolved this paradox by revealing profound developmental disturbances: an emergent ISG^+^ subpopulation indicated aberrant interferon activation; premature plasma cell differentiation suggested disrupted maturation; and biased IGLC3 expression reflected defective class switching. These alterations likely impair humoral immunity durability and quality, leading to a compromised plasma cell compartment. The coexistence of reduced plasma cells with persistent AIGAs production raises key questions: whether excessive AIGAs directly impair plasma cell survival, or whether secretion originates from a small, persistently activated cluster. Further studies are needed to establish the causal relationship between AIGAs accumulation and B-cell homeostasis.

A more interesting finding in the study is that AIGAs-positive patients have an increase in B cells expressing IFN-stimulated genes, including *ISG15*, *IFIT3*, *IFIT2*, *IFI44L*, *IFI44*, and *IFI6*. ISGs mainly exist in the cytoplasm and are induced by IFN through the JAK-STAT pathway, leading to the clearance of pathogens and maintenance of immune homeostasis (21–23). Previous studies have also found an association between the IFIT family and autoimmune diseases (23). The increased expression of IFN-stimulated genes in AIGAs-positive patients may be related to pathogen infection or immune characteristics of AIGAs. It is possible that the presence of AIGAs leads to continuous neutralization of IFN-γ and negative feedback regulation of these genes’ increase. The study suggests that B cells expressing IFN-stimulated genes may play an essential role in the disease’s pathogenesis, although we still need to determine the specific mechanisms involved.

Elevated monocyte levels in patients with stable phase immune damage were identified. IFN-γ is known to boost monocyte/macrophage antigen presentation by upregulating MHC class I/II and co-stimulatory molecules, potentially activating T cells and cytokine production, which in turn can further stimulate monocytes/macrophages (24). It’s unclear if T cell activation directly enhances monocyte/macrophage activation, warranting further research. The monocyte increase was primarily due to CD14^+^ monocytes expressing inflammatory genes like *S100A9*, *S100A8*, *IL1B*, and *CXCL8 (*25–28), often upregulated in autoimmune diseases (29, 30). Two patients exhibited eczema-like symptoms possibly linked to AIGAs-induced allergic responses. Decreased T and B cell counts in the stable phase might result from pathogen clearance, reducing antigen presentation and T and B cell numbers.

The study identified up-regulated interferon response genes in various T and B cell subsets and monocytes of AIGAs-positive patients. These genes may have a role in the disease pathogenesis. AIGAs with neutralizing ability can inhibit the JAKs-STAT signaling pathway, leading to decreased STAT-1 phosphorylation and downstream signaling. However, levels of TNF-α and IL-12 were not reduced in AIGAs-positive patients compared to healthy controls, likely due to the up-regulated interferon response genes. Further studies are needed to fully understand the complex pathogenesis of AIGAs immunodeficiency syndrome.

This study also found that HLA family genes, such as *HLA-DQB1*, *HLA-DQA1*, *HLA-DRB1*, were found to be up-regulated in most cell subsets, which is consistent with previous research and highlighting the significance of these genes in disease onset (12, 13). These genes belong to the HLA-II family, which facilitates the presentation of extracellular antigenic peptides to CD4^+^ T cells and triggers B cells to generate specific antibodies, most of the nucleated cells can express MHC-II molecules under the induction of IFN-γ (31). Recent investigations have identified associations between certain autoimmune disorders and HLA alleles. For instance, ankylosing spondylitis and reactive arthritis are linked with *HLA-B27*, multiple sclerosis is associated with *HLA-DR2*, and rheumatoid arthritis is correlated with *HLA-DR4*.

The genes *JUN*, *FOSB*, and *FOS* were significantly up-regulated in all cell subgroups. The FOS gene family consists of four genes(*FOS*、*FOSB*、*FOSL1* and *FOSL2*) that form a complex with JUN family proteins (including *c-Jun*, *JunB*, *JunD*) to create the Activator protein-1(AP-1) transcription factor (32, 33). Studies suggest that *FOS* and *JUN* are related to phosphorylation (34, 35). Research has demonstrated that the AP-1 is known for its diverse effects and plays a crucial role in numerous aspects of the immune system, including T-cell activation, Th differentiation, T-cell anergy, and exhaustion (36). Although *JUN* and *Fos* genes are highly expressed in various immune cells in AIGAs-positive patients, their role in the disease mechanism is still unclear and requires further investigation to determine whether they affect phosphorylation or immune cell regulation.

More important, our results revealed a significant down-regulation of the *IRF1* gene in all cell subsets. *IRF1* is a member of the interferon regulatory factors family and is essential for the antiviral innate immune signaling pathway (37). It binds to the upstream regulatory elements of the IFNβ1 gene and induces the expression of cytokines, IFNs, and ISGs (38). Furthermore, both IFN-γ-dependent macrophage immunodeficiency and IFN-α/β-dependent immunodeficiency result in an increase in pathogen susceptibility due to the absence of *IRF1 (*39–41). These findings suggest that *IRF1* plays a crucial role in combating pathogen infection and the low expression of *IRF1* may lead to increased susceptibility to pathogen infection, which requires further investigation.

KEGG and GO pathway enrichment analyses were conducted to identify differentially expressed genes. The results revealed that these genes were significantly enriched in infectious diseases, T cell differentiation, antigen presentation, and TNF signaling pathways. Although further studies using cell or animal experiments are required to fully understand the specific mechanisms underlying these pathways, our findings provide valuable insights into the immunological mechanisms involved in disease pathogenesis.

In this study, scRNA-seq was used to depict the peripheral immune landscape of AIGAs immunodeficiency syndrome. T cells, B cells, monocytes, and NKT cells were identified as the main immune cells in PBMCs of AIGAs immunodeficiency syndrome. Increased Th1 cells and ISG_B cells, which may be related to disease pathogenesis. The function of T cells and the production of IFN-γ in AIGAs immunodeficiency syndrome are normal, and the inactivation of IFN-γ caused by the production of AIGAs leads to immunodeficiency in patients. The decrease of plasma cells may be related to the depletion of plasma cells caused by the production of AIGAs. The proportion of immune cells in different disease phases is inconsistent. It was also found that the occurrence of diseases was related to multiple genes, and some immune-related metabolic pathways were identified. The above findings are of great significance for further understanding the immune characteristics and pathogenesis of the disease, and also provide data basis for subsequent targeted therapy.

This study, despite its multi-approach characterization of AIGAs immunodeficiency, has limitations. The sample size was small, especially for healthy controls, limiting statistical power. Lacking longitudinal data, we could not establish causality between immune changes and disease progression. Furthermore, key serum proteins like IFN-γ showed no significant change, indicating transcriptional findings may not reflect systemic protein levels and require functional validation. Finally, the role of expanded novel subsets like ISG^+^ B cells remains unclear and needs further investigation.

In summary, this first single-cell atlas defines AIGAs immunodeficiency syndrome as a Th1-skewed, IFN-γ-driven disorder orchestrated by CD4^+^ Tem–CD14^+^ monocyte crosstalk. The heterogeneous immune landscape features T-cell activation, expansion of Th1 and ISG^+^ B cells, and loss of memory/plasma B cells—likely explaining autoantibody production. Pseudotemporal analysis confirms skewed T- and B-cell differentiation, and these dynamic immunopathological signatures are underpinned by polygenic upregulation of interferon and HLA pathways, offering a mechanistic basis for targeted therapy.

## Data Availability

The raw sequence data reported in this paper have been deposited in the Genome Sequence Archive (42) in National Genomics Data Center (43), China National Center for Bioinformation / Beijing Institute of Genomics, Chinese Academy of Sciences (GSA-Human: HRA016092) that are publicly accessible at https://ngdc.cncb.ac.cn/gsa-human.

## Supplementary material

Supplementary Figure S1 Flow cytometric profiling of lymphocyte subsets in a representative AIGAs immunodeficiency patient. The figure shows the gating and frequency of **(A)** Th1 (IFN-γ^+^CD4^+^, 25.15%), **(B)** Th17 (IL-17^+^CD4^+^, 4.03%), and **(C)** Treg (CD25^+^FOXP3^+^, 6.50%) cells among CD4^+^ T cells; and among B cells (CD19^+^, 10.27% of lymphocytes): (D) marginal zone-like (CD27^+^IgD^+^, 4.45%), **(E)** memory (CD27^+^CD38^dim^, 13.85%), and **(F)** class-switched memory (CD27^+^IgD^–^IgM^–^, 5.63%) subsets. Data are from 1,880 total lymphocytes.

Supplementary Figure S2 The expression of gene IGHA1, IGHA2, and IGHE in AIGAs-positive patients and healthy controls. **(A)** The expression of gene IGHA1. **(B)** The expression of gene IGHA2. **(C)** The expression of gene IGHE.

Supplementary Figure S3 Pseudotime analysis of T cells. **(A)** Pseudotime trajectory of T-cell differentiation inferred by Monocle 2. **(B)** Monocle2 pseudotime plot colored by Seurat-annotated cell types.

Supplementary Figure S4 Pseudotime analysis of CD4^+^ T cells. **(A)** Pseudotime trajectory of CD4^+^ T cell differentiation inferred by Monocle 2. **(B)** Monocle 2 pseudotime plot colored by Seurat-annotated CD4^+^ T cell subtypes.

Supplementary Figure S5 Patient-derived CD4^+^ T cells accumulate at pseudotime node 4 and up-regulate LYZ, S100A8, and S100A9 in the CD4_Tem subset.

Supplementary Figure S6 Pseudotime trajectory of B cells differentiation inferred by Monocle 2.
