# Supplementary figures and images for: Upregulation of interferon-γ activation in patients with anti-interferon-γ autoantibodies immunodeficiency syndrome: insights from single-cell analysis
Source: Front Immunol. 2026 Feb 3;16:1659383. doi: 10.3389/fimmu.2025.1659383 (PMC12909572; doi:10.3389/fimmu.2025.1659383)

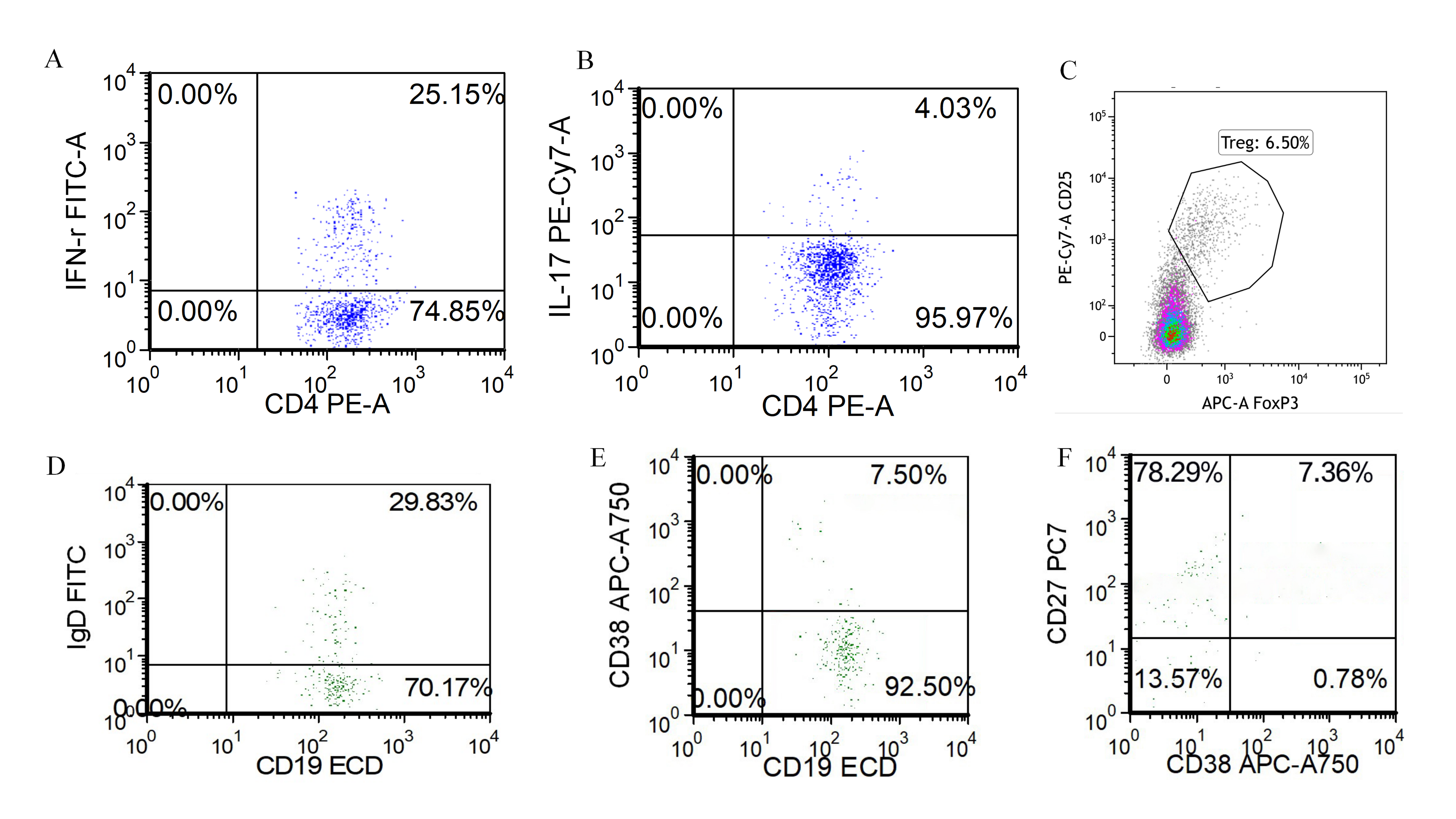

Supplement: Supplementary Figure S1 — Flow cytometric profiling of lymphocyte subsets in a representative AIGAs immunodeficiency patient. The figure shows the gating and frequency of (A) Th1 (IFN-γ+CD4+, 25.15%), (B) Th17 (IL-17+CD4+, 4.03%), and (C) Treg (CD25+FOXP3+, 6.50%) cells among CD4+ T cells; and among B cells (CD19+, 10.27% of lymphocytes): (D) marginal zone-like (CD27+IgD+, 4.45%), (E) memory (CD27+CD38dim, 13.85%), and (F) class-switched memory (CD27+IgD–IgM–, 5.63%) subsets. Data are from 1,880 total lymphocytes. [file Image1.tif]

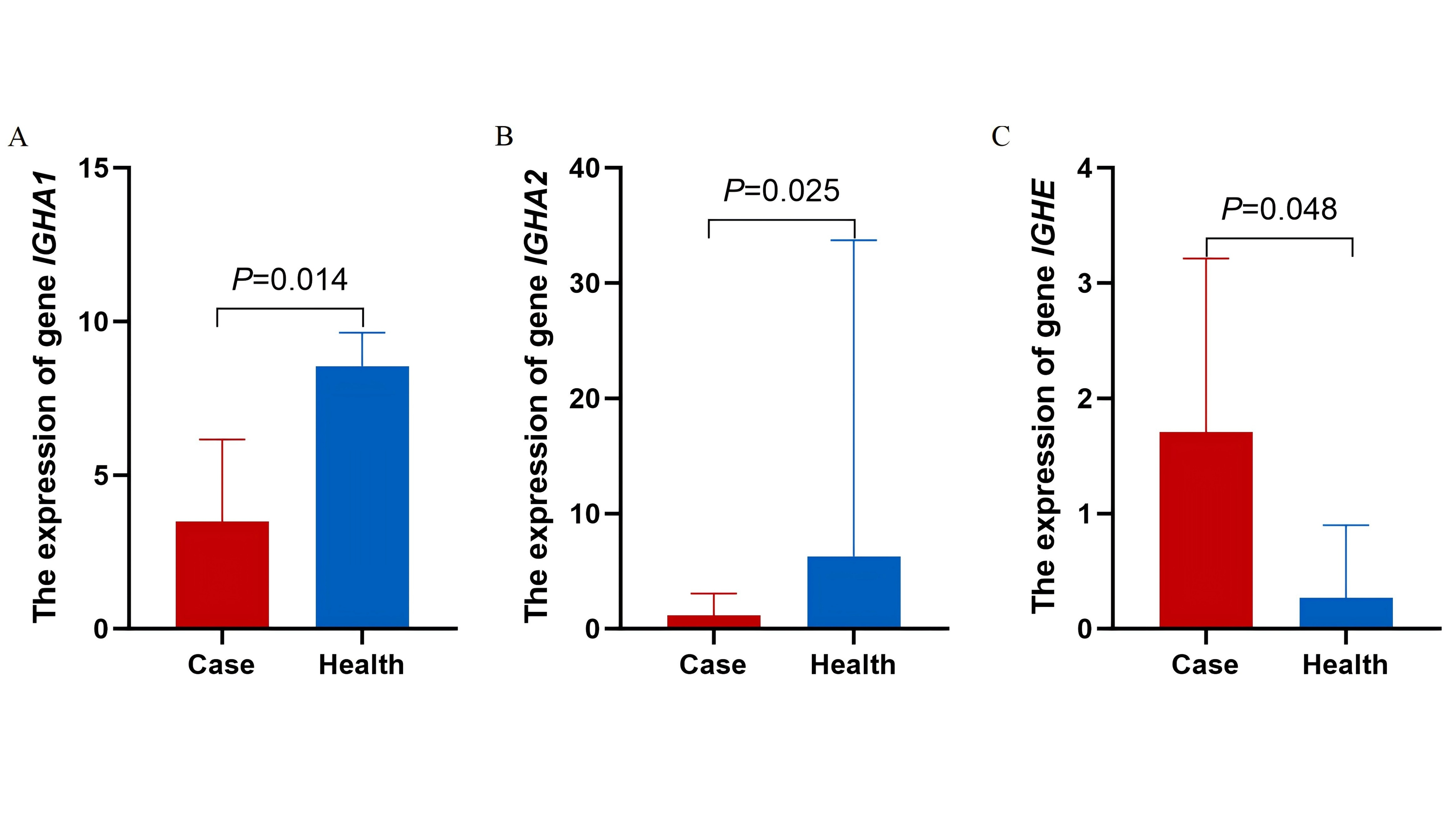

Supplement: Supplementary Figure S2 — The expression of gene IGHA1, IGHA2, and IGHE in AIGAs-positive patients and healthy controls. (A) The expression of gene IGHA1. (B) The expression of gene IGHA2. (C) The expression of gene IGHE. [file Image2.tif]

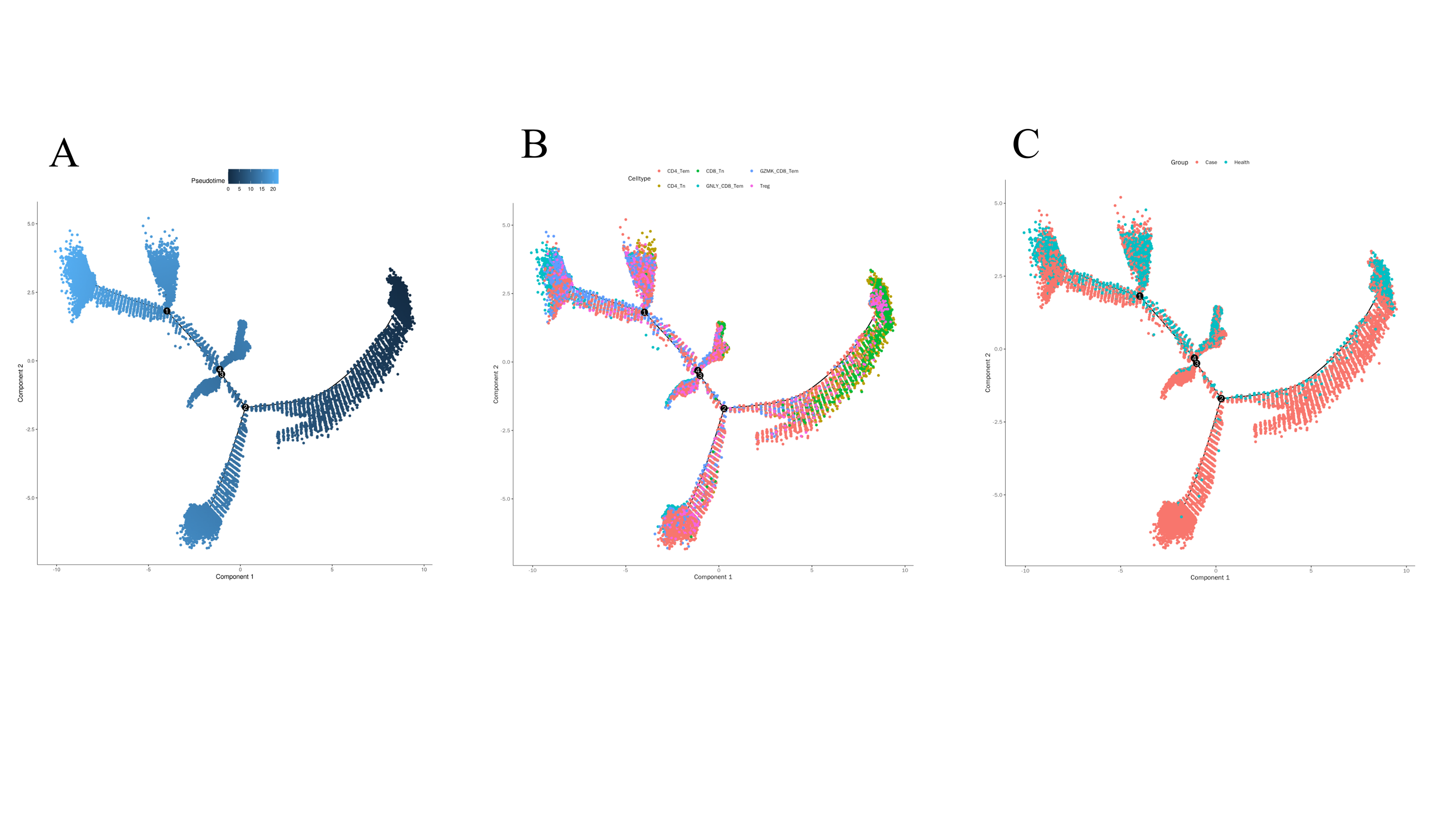

Supplement: Supplementary Figure S3 — Pseudotime analysis of T cells. (A) Pseudotime trajectory of T-cell differentiation inferred by Monocle 2. (B) Monocle2 pseudotime plot colored by Seurat-annotated cell types. [file Image3.tif]

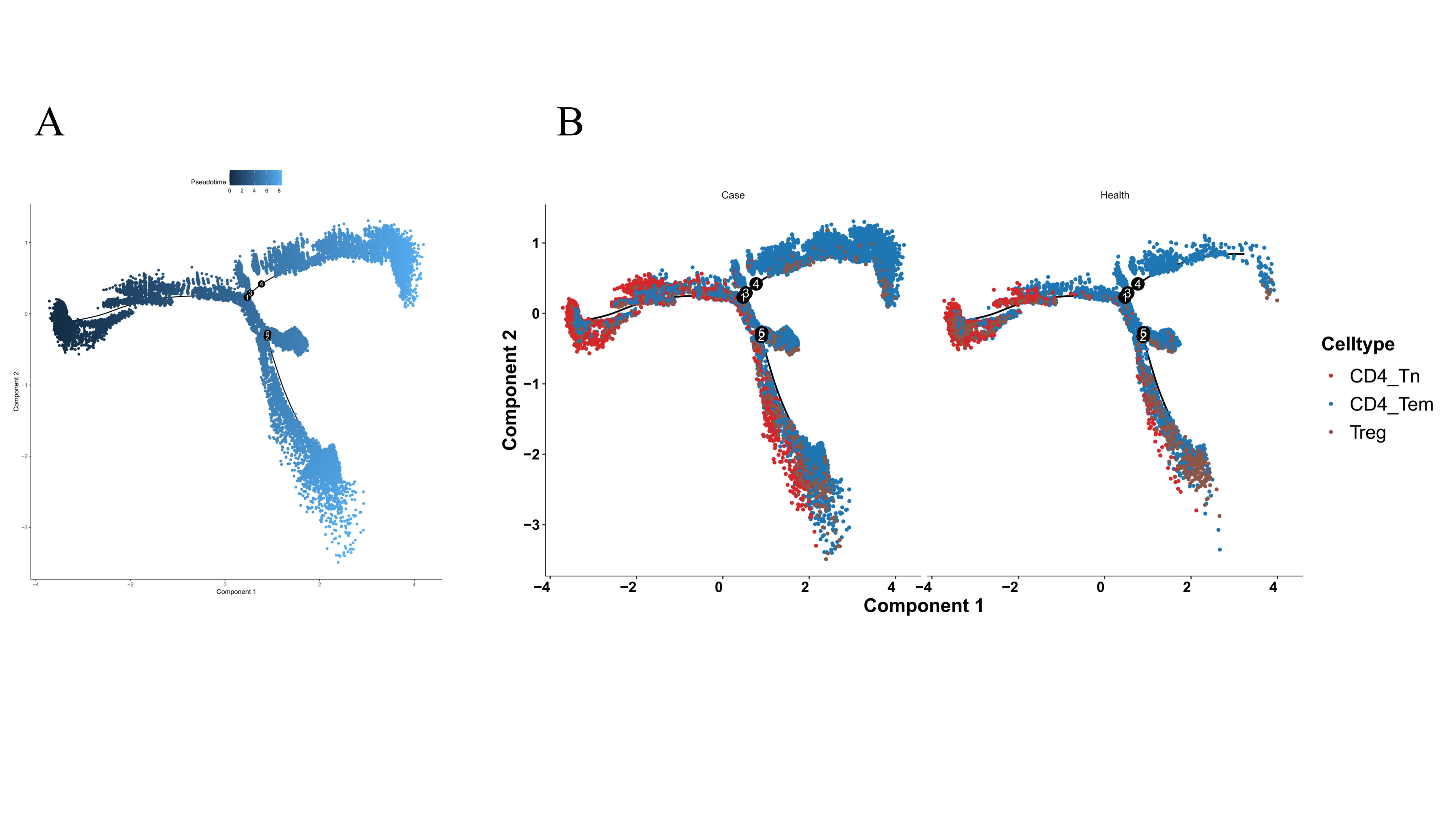

Supplement: Supplementary Figure S4 — Pseudotime analysis of CD4+ T cells. (A) Pseudotime trajectory of CD4+ T cell differentiation inferred by Monocle 2. (B) Monocle 2 pseudotime plot colored by Seurat-annotated CD4+ T cell subtypes. [file Image4.tif]

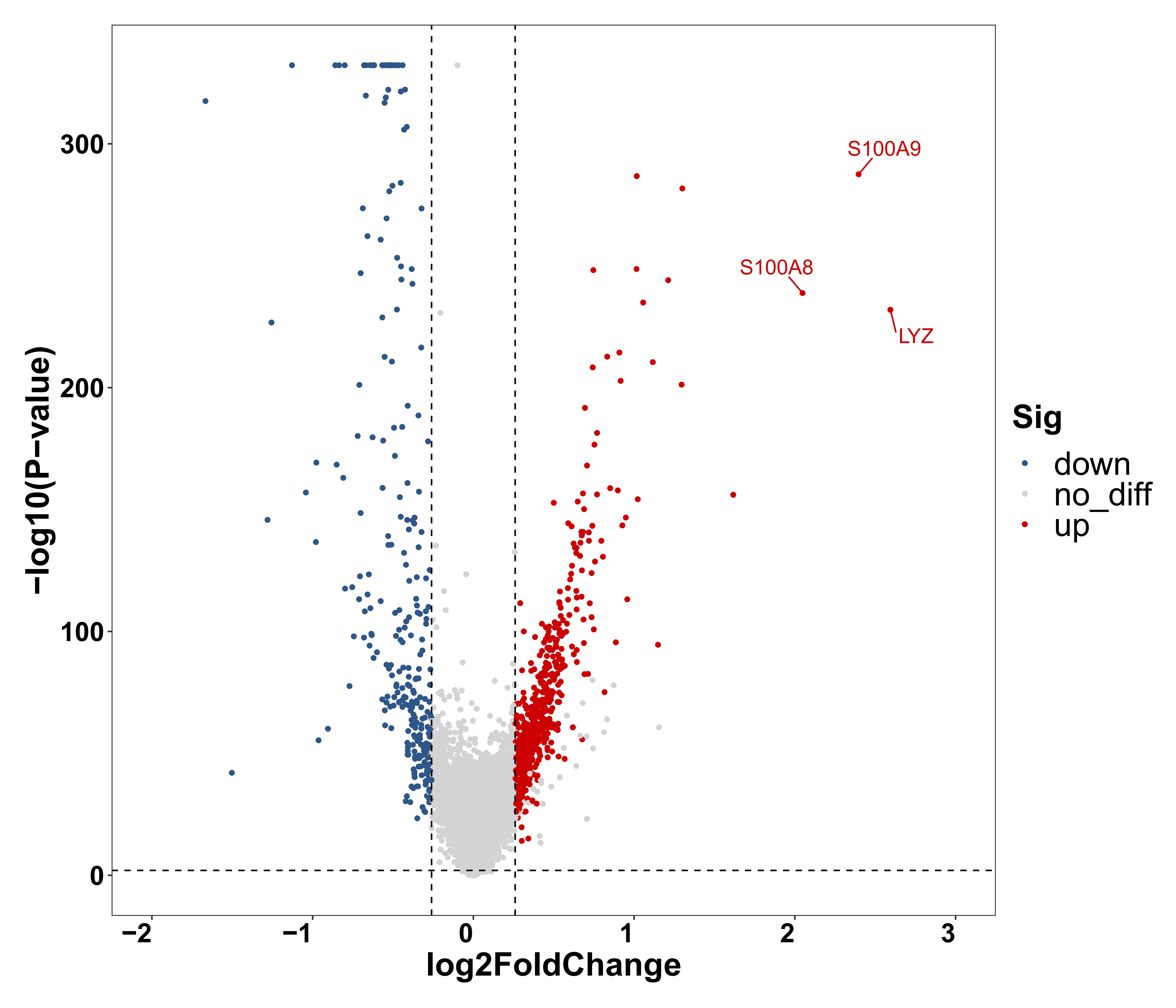

Supplement: Supplementary Figure S5 — Patient-derived CD4+ T cells accumulate at pseudotime node 4 and up-regulate LYZ, S100A8, and S100A9 in the CD4_Tem subset. [file Image5.png]

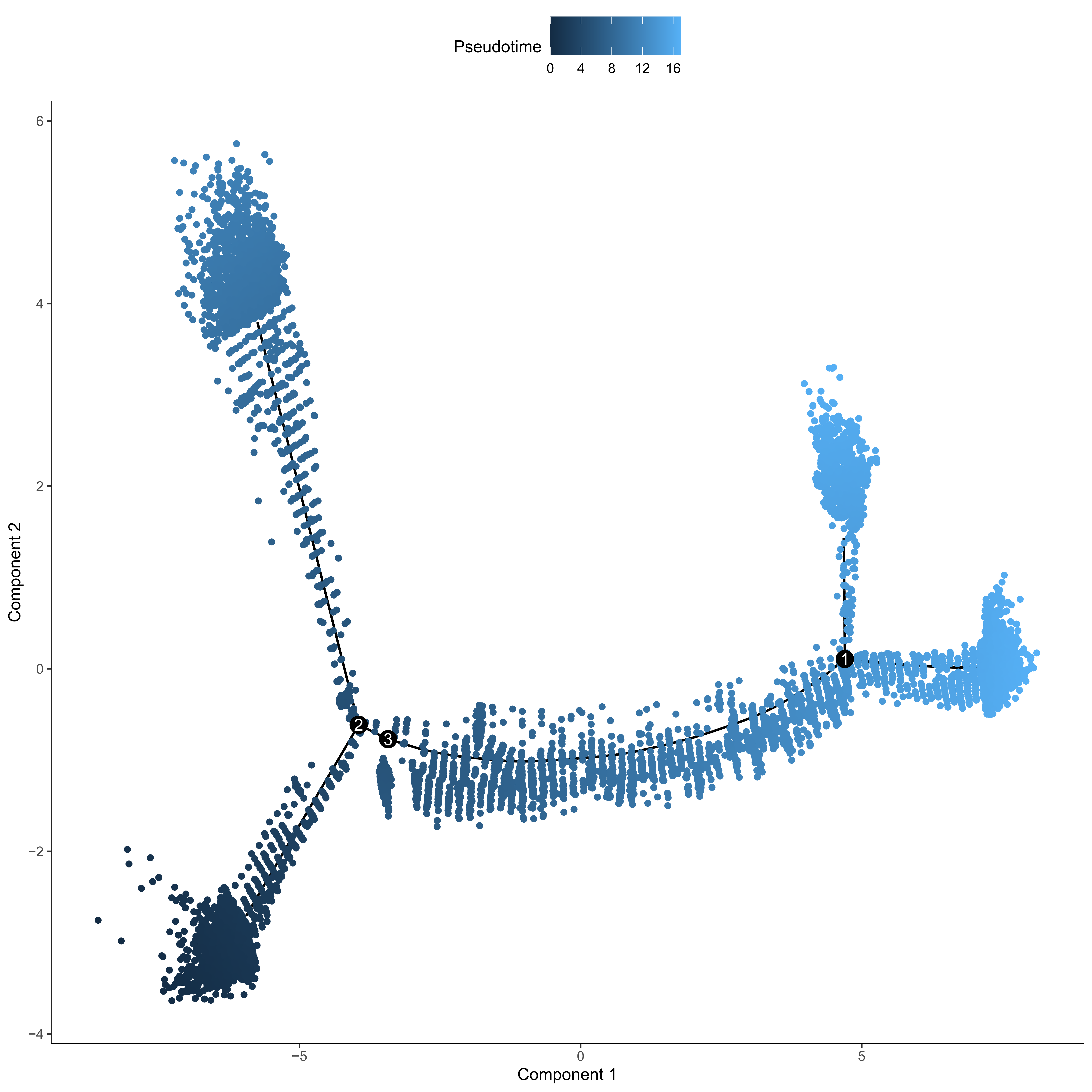

Supplement: Supplementary Figure S6 — Pseudotime trajectory of B cells differentiation inferred by Monocle 2. [file Image6.png]
