# Supplementary material for: Upregulation of interferon-γ activation in patients with anti-interferon-γ autoantibodies immunodeficiency syndrome: insights from single-cell analysis
Source: Front Immunol. 2026 Feb 3;16:1659383. doi: 10.3389/fimmu.2025.1659383 (PMC12909572; doi:10.3389/fimmu.2025.1659383)
Supplement: Supplementary file 7 [file Table1.docx]

| Supplementary Table 1.Baseline demographic and clinical characteristics of the study subjects | | | | | | | | | | | | |
| --- | --- | --- | --- | --- | --- | --- | --- | --- | --- | --- | --- | --- |
| Subject ID | Gender | Age (years) | IgE (IU/ml) | IgG (g/L) | IgG4 (g/L) | ESR（mm/h） | CRP（mg/dl） | WBC (109/L) | Neu (109/L) | Lym (109/L) | Eos (109/L) | Mon (109/L) |
| P1 | Male | 39 | 481.3 | 28.6 | 2.7 | 121.0 | 127.9 | 8.0 | 5.3 | 1.3 | 0.9 | 0.4 |
| P2 | Male | 53 | 1631.4 | 29,6 | - | 87.0 | 70.0 | 30.6 | 24.8 | 3.6 | 0.8 | 1.3 |
| P3 | Male | 45 | 126.3 | 46.4 | 0.9 | 63.0 | 23.05 | 8.7 | 6.0 | 1.7 | 0.5 | 0.5 |
| P4 | Female | 25 | 90.9 | 12.9 | 3.3 | 42.0 | 19.0 | 13.9 | 9.1 | 3.4 | 0.4 | 0.9 |
| P5 | Male | 53 | 96.4 | 9.0 | - | 34.0 | 4.0 | 15.6 | 11.9 | 2.8 | 0.1 | 0.8 |
| P6 | Male | 66 | 135.0 | 17.0 | 3.3 | 5.0 | 0.8 | 16.1 | 4.6 | 2.8 | 7.8 | 1.5 |
| P7 | Male | 38 | 205.0 | 39.7 | 8.1 | 87.0 | 120.3 | 10.3 | 7.7 | 1.6 | 0.4 | 0.7 |
| P8 | Female | 46 | 20.7 | 16.6 | 0.4 | 140.0 | 102.0 | 9.1 | 8.6 | 0.4 | 0.1 | 0.1 |
| H1 | Male | 50 | - | - | - | - | - | - | - | - | - | - |
| H2 | Male | 37 | - | - | - | - | - | - | - | - | - | - |
| H3 | Female | 34 | - | - | - | - | - | - | - | - | - | - |
| IgE: Immunoglobulin E; IgG:Immunoglobulin G; IgG4:mmunoglobulin G4; CRP:C-Reactive Protein; WBC:White Blood Cell Count; Neu:Neutrophils; Lym:Lymphocytes; Eos:Eosinophils; Mon:Monocytes | | | | | | | | | | | | |
